# Exploring the retention of soluble Fas protein in kidney dysfunction and its link to inflammation: a systematic review and meta-analysis

**DOI:** 10.1590/2175-8239-JBN-2025-0146en

**Published:** 2026-03-09

**Authors:** Beatriz Moreira Silva, Giovana Irikura Cardoso, Priscila Giusti Lázaro, Bárbara Formaggio Domingues, Isabele Pardo, Carolina Sponton, Miguel Angelo Goes

**Affiliations:** 1 Escola Paulista de Medicina, Universidade Federal de São Paulo, São Paulo, SP, Brazil.; 2 Faculdade Israelita de Ciências da Saúde Albert Einstein, São Paulo, SP, Brazil.

**Keywords:** Chronic Kidney Disease, Acute Kidney Injury, Soluble Fas, Inflammation, Uremic Solute

## Abstract

**Introduction:**

According to recent studies, serum soluble Fas (sFas) levels are related to inflammatory markers, cardiovascular disease, anemia, and kidney dysfunction. The present study analyzes the association between sFas, inflammatory markers, and kidney dysfunction.

**Methods:**

This meta-analysis was conducted using the R program, along with a systematic review that employed the Newcastle-Ottawa Scale and the Joanna Briggs Institute guidelines to assess risk of bias in observational studies. The PubMed, MEDLINE, and SciELO electronic databases were used. Search filters were applied to include all articles in English. Heterogeneity was assessed using Cochran’s Q test, τ^2^ (tau-squared), and the I^2^ statistic, and a random-effects model (DerSimonian-Laird method) was applied due to the presence of moderate heterogeneity among the included cohort studies.

**Results:**

The systematic review and meta-analysis comprised 24 articles, which presented a significant risk of bias. Among the selected cohort studies, 1,449 patients had kidney dysfunction. Of these, 512 (57%) were men. The mean serum creatinine, IL-6, C-reactive protein, and sFas values (8.635 pg/mL and 3.206 pg/mL) were higher in patients with kidney dysfunction. There were also positive correlations between serum sFas, IL-6 levels, and creatinine. These findings suggest a potential role of sFas in inflammation and the progression of kidney diseases.

**Conclusion:**

Our study demonstrated that sFas is a uremic retention solute associated with inflammation. Further research is crucial to confirm sFas as a biomarker or its role as a uremic toxin.

## Introduction

Renal dysfunction in both acute kidney injury (AKI) and chronic kidney disease (CKD) is marked by decreased kidney function and increased levels of uremic retention solutes^1,2,3,4^.

Soluble Fas (sFas) is a protein derived from the CD95^+^ receptor (mFas)^5^. Unlike the CD95^+^ receptor, which is anchored in the cell membrane, sFas lacks a transmembrane domain due to either metalloproteinase cleavage of mFas or alternative splicing of the CD95^+^ pre-mRNA. While mFas functions as a transmembrane glycoprotein and activates apoptosis and other pathways through the Fas ligand (FasL), sFas may interfere with mFas signaling^5,6,7,8,9,10,11^.

Elevated serum sFas levels are associated with cardiovascular disease, anemia, and inflammation in CKD^12,13,14,15,16,17,18^. Serum sFas levels also significantly correlate with anemia and inflammation in AKI^19,20^. The critical relationship between sFas and inflammation in kidney dysfunction might help healthcare professionals and researchers understand and develop effective management strategies for these severe conditions.

Recognizing that inflammation influences cardiovascular disease and anemia in CKD and AKI, we conducted this study to better understand the potential relationship between sFas, kidney function markers, and inflammation in kidney dysfunction, such as C-reactive protein (CRP) and interleukin-6 (IL-6).

## Methods

This study is a systematic review and meta-analysis. We thoroughly searched the PubMed, MEDLINE, and SciELO platforms using MeSH terms (Supplementary Material). The authors carefully selected articles for inclusion in the study, adhering to strict criteria. Experimental articles, meta-analyses, and review articles were excluded. In contrast, analyses published in English that utilized MeSH terms and query filters for observational studies were included (Supplementary Annex). The last search was conducted on January 20, 2024.

**Figure 1:**
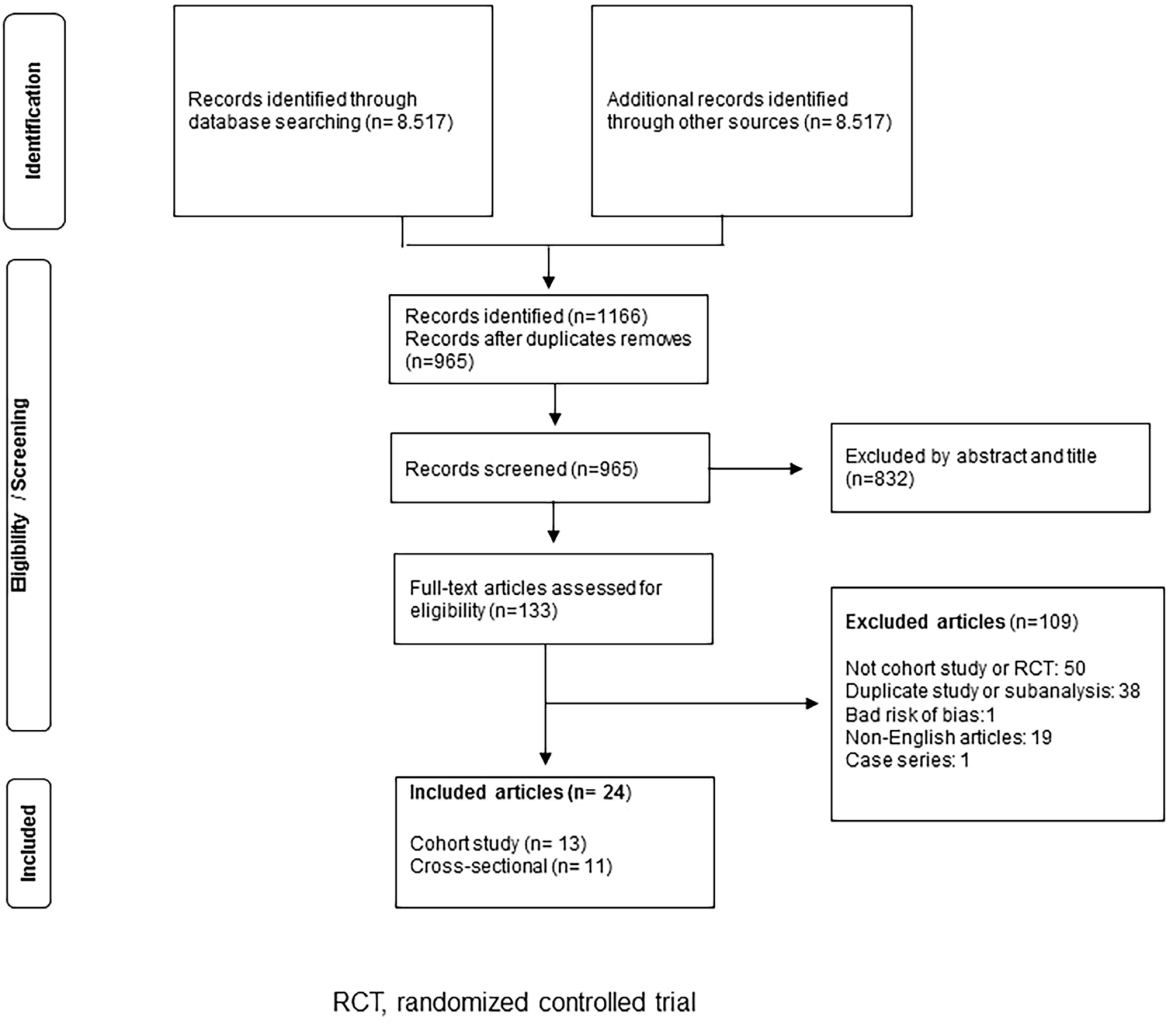
Summary of evidence search and selection.

We used the Newcastle-Ottawa Scale (NOS) to assess and filter the risk of bias in the cohort studies included in this systematic review. The NOS evaluates the quality of non-randomized studies based on selection, comparability, and outcome analysis. Of a total of 21 cohort articles, only 14 were selected for the analysis, and among these, one article was classified as low quality and was removed from the study, resulting in 13 cohort articles selected for the systematic review. For cross-sectional studies (n = 13), we used the Joanna Briggs Institute (JBI) checklist, a validated tool for observational studies. Two researches were excluded due to a high risk of bias, leaving 11 cross-sectional studies for analysis. After screening and eligibility assessment, 24 studies met the inclusion criteria and were selected for analysis (Figure 1).

Risk of bias and quality of evidence were assessed independently by two reviewers. Any disagreements were resolved by consensus with a third reviewer. The quality and risk of bias assessments directly guided study inclusion and interpretation: only cohort studies with moderate-to-high NOS scores and cross-sectional studies classified as low risk of bias by the JBI were retained for synthesis. These assessments were applied as screening tools and used to contextualize the strength of the evidence in the Results and Discussion sections.

A total of 13 articles were included in the systematic review. However, the quantitative synthesis was restricted to 8 cohort studies, since they provided statistically comparable data regarding the association between sFas, serum creatinine, and inflammatory markers, suggesting relationships among these variables. The remaining cross-sectional studies were analyzed descriptively and excluded from the pooled analysis to minimize methodological inconsistencies.

All statistical analyses were performed in the R software (version 4.1.2) using the “meta” and “metafor” packages. A random-effects model (REM) was employed to account for between-study variability, ensuring that study weights were calculated according to variance rather than being equally distributed. Effect sizes were expressed as odds ratios (OR) or standardized mean differences (SMD), both with 95% confidence intervals (CIs).

In addition, heterogeneity was assessed using Cochran’s Q test (χ^2^, with p < 0.10 indicating heterogeneity), the inconsistency index (I^2^, expressed as the percentage of total variability not attributable to chance), and τ^2^ (between-study variance estimate). These measures, together with pooled effect sizes and study weights, are explicitly displayed in the forest plots. To explore potential publication bias, we generated a funnel plot (Supplementary Figure 1). In the main quantitative synthesis of the 8 cohort studies, Egger’s test was applied; however, the results were interpreted with caution, given the limited number of articles. Subgroup analyzes by clinical phenotype (CKD vs. AKI) were considered but not performed, as the small number of researches in each category would not support robust comparisons.

Each study contributed a distinct statistical weight to the pooled estimate according to its sample size and variance, as displayed in the forest plot. The forest plot also presents individual study estimates with 95% CIs, along with study weights under the random-effects model.

According to Resolution CNS 510/2016 (Section IV), research carried out exclusively using scientific texts for a scientific literature review is neither registered nor evaluated by the ethics committee system (CEP/CONEP). Thus, the present study can be conducted without local approval and processing. Even so, we have indexed our project in PROSPERO, a respected international database for systematic review protocols, reinforcing methodological transparency.

## Results

We performed article selection ethically and systematically (Figure 1). Firstly, there were 8,517 articles. After rigorous screening, removal of duplicates, and peer review, we selected 965 researches. To further refine this selection, we excluded studies that were not published in English and those that did not meet our predefined inclusion criteria. Even though we eliminated researches that did not meet the requirements for study design, randomized controlled trials, or appropriate sub-analyses, we ensured that only relevant and high-quality research was included.

This resulted in the exclusion of 941 articles. However, 24 articles were deemed the most relevant for inclusion in the systematic review and meta-analysis, comprising 13 cohort studies of good quality and 11 cross-sectional studies with a low risk of bias. The NOS and the JBI guidance were used not only for methodological quality assessment but also played a key role in guiding the interpretation of the review findings. Our review began with a detailed analysis of the risk of bias in cohort studies (Table 1) and cross-sectional studies (Table 2). It was essential to maintain the integrity of our review process. Data from the cohort studies allowed us to correlate several variables with patient groups, both those with and without renal dysfunction. Notably, 64% of the individuals analyzed in the studies exhibited some degree of renal dysfunction. Among the individuals included, 836 (57%) were men, and the mean age of patients with renal dysfunction—a critical factor in their management—was 46 years.

**Table 1:** Risk of bias of the cohort studies

Authors	Selection				Comparability	Outcome		
	Representativeness of the exposed cohort	Selection of the non-exposed cohort	Ascertainment of exposure	Demonstration that outcome of interest was not present at start of study	Comparability of cohorts on the basis of study design or controlled analysis for confounders	Assessment of outcome	Was follow-up long enough for outcomes to occur?	Adequacy of follow-up of cohorts
GÓES et al. (2013)^1^	*	*	*	*	**	*	*	*
GÓES et al. (2010)^21^	*	*	*	*	*	*	*	*
PERIANAYAGAM et al. (2000)^2^	*	*	*	*	**	*	*	*
KORKES et al. (2013)^9^	*	*	*	*	*	*	*	*
SANO et al. (1998)^11^	*	*	*	*	**	*	*	*
SHOU et al. (1999)^10^	*	*	*	*	**	*	-	*
ADLY et al. (2016)^22^	*	*	*	*	**	*	-	*
BHATRAJU et al. (2017)^13^	*		*	*	**	*	-	*
DALBONI et al. (2003)^16^	*	*	*	*	**	*	*	*
TROYANOV et al. (2003)^23^	*		*	*	*	-	*	*
NONOMURA et al. (2000)^24^	*	*		*	*	*	*	*
EL-ADROUDY et al. (2000)^8^	*	*	*	*	**	*	*	-
ZWIECH et al. (2013)^25^		*	*	*	**	*	*	*

**Table 2:** Risk of bias in the cross-sectional studies

Authors	Were the criteria for inclusion in the sample clearly defined?	Were the study subjects and the setting described in detail?	Was the exposure measured in a valid and reliable way?	Were objective, standard criteria used for measurement of the condition?	Were confounding factors identified?	Were strategies to deal with confounding factors stated?	Were the outcomes measured in a valid and reliable way?	Was appropriate statistical analysis used?
MASRI et al. (2000)^15^	Y	Y	Y	Y	Y	Y	Y	Y
DALBONI et al. (2008)^5^	Y	Y	Y	Y	Y	Y	Y	Y
DOUNOUSI et al. (2012)^18^	Y	Y	Y	Y	Y	Y	Y	Y
MORILLAS et al. (2012)^26^	Y	Y	Y	Y	Y	Y	Y	Y
SATO et al. (2000)^27^	Y	Y	Y	Y	Y	Y	Y	Y
TOMIYAMA et al. (2006)^30^	Y	Y	Y	Y	Y	Y	Y	Y
BABA et al. (2004)^28^	Y	Y	Y	Y	N	Y	Y	Y
D’ANDREA et al. (2020)^32^	Y	Y	Y	Y	Y	N	Y	Y
RESCH et al. (2021)^33^	Y	Y	Y	Y	Y	N	Y	Y
NIEWCZAS et al. (2008)^29^	Y	Y	Y	Y	Y	Y	Y	Y
AMMIRATI et al. (2006)^31^	Y	Y	Y	Y	Y	Y	Y	Y

Regarding serum data, patients with renal dysfunction had a mean serum creatinine level of 2.8 mg/dL, whereas those without renal dysfunction had a mean level of 0.7 mg/dL. We also obtained serum sFas values, finding that the mean serum value for patients with renal dysfunction was 8.635 pg/mL, compared with 3.206 pg/mL in the group without renal dysfunction. The mean serum CRP value for the group with renal involvement was 0.035 mg/dL, which did not differ significantly from the value of 0.034 mg/dL observed in the group of study subjects without renal dysfunction. In addition, mean serum IL-6 levels were notably elevated in patients with renal dysfunction (Table 3).

**Table 3:** General information on all cohort studies

General information	Renal involvement	No renal involvement
**Patients**	1449	815
**Women (%)**	512 (43)	365 (44)
**Men (%)**	836 (57)	459 (56)
**Average age of the patients**	46	35
**Average serum creatinine (mg/dL)**	2.8	0.7
**Average serum sFas (pg/mL)**	8635	3206
**Average serum CRP (mg/dL)**	0.0359	0.0345
**Average serum IL-6 (pg/mL)**	193.4	29.7

Abbreviations – sFas: Soluble Fas; CRP: C-Reactive Protein; IL-6: Interleukin-6.

Notes – Average values were calculated by the authors using data extracted from the previously published studies^1,2,8,9,10,11,13,16,21,22,23,24,25^.

**Figure 2:**
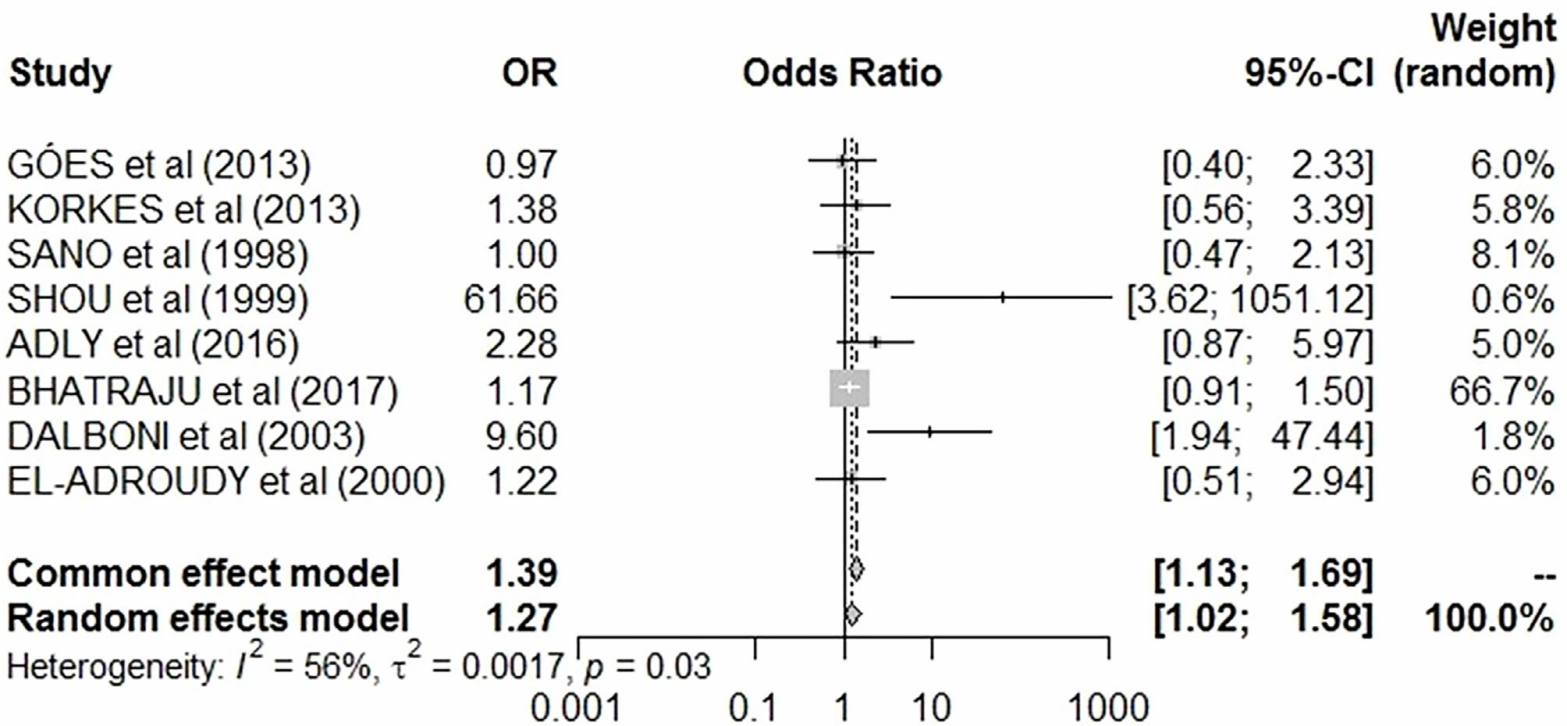
Forest plot of the association between sFas and creatinine.

A total of 13 cohort studies were included in the systematic review. Of these, only 8 cohort studies were eligible for the meta-analysis, as they reported comparable and statistically significant data on the association between sFas levels and kidney dysfunction. The 11 cross-sectional studies were synthesized narratively due to methodological heterogeneity. In the quantitative synthesis of the 8 cohort studies, patients with impaired kidney function exhibited significantly higher circulating levels of sFas compared with controls (pooled OR = 1.27, 95% CI = 1.02–1.58, p = 0.03). Between-study heterogeneity was moderate (Cochran’s Q = 14.2, p = 0.048; I^2^ = 56%; τ^2^ = 0.0017), as displayed in the forest plot (Figure 2).

Abbreviations – OR: odds ratio; 95% CI: 95% confidence interval; I^2^: inconsistency index (heterogeneity across studies); τ^2^: between-study variance (tau-squared); p: statistical significance value.

Inflammatory markers, particularly IL-6, were reported in 6 studies. Although some individual analyses suggested a positive correlation between IL-6 and sFas, only two studies provided quantitative estimates, which were insufficient to allow for a pooled analysis.

Publication bias was evaluated for the 8 cohort studies. Visual inspection of the funnel plot (Supplementary Figure 1) revealed approximate symmetry, with no major evidence of small-study effects. The Egger’s regression test (p = 0.12) did not indicate significant publication bias; however, interpretation must be cautious given the limited number of studies (< 10 studies).

No additional sensitivity or subgroup analyses (e.g., CKD vs. AKI) were performed due to the small number of available studies in each category. This limitation is acknowledged, as combining CKD and AKI data may reduce phenotype-specific interpretability.

In our statistical analysis, we extracted information on the relationship between sFas and the inflammatory marker CRP. The meta-analysis demonstrated a statistically significant association between sFas and CRP values in patients with renal dysfunction [OR = 0.72, 95% CI = 0.70–0.73; p = 0.001]. However, only three studies provided sufficient data for this calculation, which limits the robustness and generalizability of this finding.

## Discussion

Our research establishes a clear link between serum sFas levels and creatinine levels. Since serum creatinine is a vital marker for assessing kidney function in patients with kidney diseases, it is evident that sFas functions as a uremic retention solute. Additionally, we found a significant correlation between sFas and serum levels of IL-6, a key pro-inflammatory cytokine. Our findings facilitate the understanding of kidney dysfunction and provide valuable insights to improve research efforts and patient management strategies for complications related to kidney dysfunction.

We analyzed 13 cohort studies that met high selection criteria, each consisting of well-defined and comprehensive cohorts. Notably, the group without kidney dysfunction was drawn from the same research cohort as those with AKI or CKD, enhancing the reliability of our results and contributing significantly to the field.

The essential assessment criteria were clearly defined to ensure adequate comparability of data, maintaining consistency in how data were presented across articles. While some studies focused exclusively on specific subgroups, others did not clarify whether the group without kidney dysfunction was explicitly designed for that study, unlike the kidney dysfunction group. However, these inconsistencies did not significantly affect the overall quality of the findings. Eleven cross-sectional studies were included descriptively due to methodological heterogeneity, while the quantitative synthesis was restricted to the 8 cohort studies that reported comparable and statistically significant data.

We addressed heterogeneity through standard statistical tests and applied a random-effects model, which allowed for more robust pooled estimates. Although Egger’s test was interpreted with caution given the limited number of reviews, the funnel plot suggested a low risk of publication bias. Each study was weighted by the inverse variance method, ensuring that more precise data contributed proportionally to the overall effect.

On the other hand, eleven cross-sectional studies were selected based on the comprehensiveness and quality of the information concerning the inclusion criteria. The selection process was conducted in a systematic manner, considering factors such as the relevance of the topic, a clear association between sFas and kidney dysfunction through well-defined objectives, appropriate measurement of exposure, the rigor of statistical analyses, and other relevant methodological considerations.

In the present study, when we analyzed researches of patients with kidney dysfunction—either AKI or CKD—we observed that serum levels of sFas were higher in patients with kidney dysfunction compared with individuals without kidney dysfunction. Our group had previously reported that serum levels of sFas negatively correlated with creatinine clearance in patients with CKD and were higher in patients with advanced CKD requiring maintenance dialysis^21^. A study by Dalboni et al. found that serum levels of sFas were significantly higher in individuals with kidney dysfunction than in those without it. Patients with CKD and cardiovascular disease had even higher sFas levels. There was an inverse correlation between serum sFas levels, creatinine clearance, and the dosage of recombinant human erythropoietin. Additionally, sFas levels had a positive relationship with CRP levels. These findings highlight the potential solute retention of sFas in kidney dysfunction, suggesting that sFas may be a biomarker for inflammation and disease-related conditions^16^.

Our analysis identified a statistically significant association between serum sFas and CRP levels in patients with renal dysfunction. However, this result was based on data extracted from only 3 studies, which limits the strength and generalizability of the finding^16,21,22^. We also found significant differences in the analyses of IL-6 levels in the articles, which showed that serum IL-6 levels were higher in AKI and CKD patients^19,20,23^. Serum levels of IL-6 showed a trend toward increasing with the deterioration of kidney function^24^. This also suggests that serum sFas could be a key marker of inflammation in uremic patients. Even though multiple studies addressed inflammatory markers, with IL-6 receiving particular attention, the limited availability of quantitative data precluded a combined statistical analysis. While some findings suggested a possible positive association between IL-6 and sFas, this relationship should be considered preliminary and hypothesis-generating.

Our previous studies have demonstrated that serum sFas levels are higher in patients with CKD than in those without kidney dysfunction^15,17,18,19,20,21^. This trend is also evident in patients with AKI, who show higher serum sFas levels than those without kidney dysfunction^19,20^. Furthermore, a strong connection was found between sFas levels, inflammation, and anemia in kidney diseases^15,17,19,20,21^. Since serum levels of sFas are higher in patients with renal dysfunction than in healthy individuals, this highlights the importance of sFas as a potential uremic retention solute.

This study has several limitations. First, there was a lack of articles explicitly investigating the relationship between sFas and renal dysfunction in cohort and cross-sectional studies. Although the included research provided valuable data for analyzing the clinical profiles of patients with renal conditions, the limited number of studies on this topic hampers our ability to establish robust and meaningful correlations between variables. Second, the interpretation of Egger’s regression test was limited by the small number of studies included, since this method requires a larger sample size to reliably detect publication bias. The analysis of IL-6 was also limited by the small number of articles reporting this marker, which reduces the robustness of the findings. Finally, we included both AKI and CKD populations in a pooled analysis. Although we attempted to stratify outcomes by clinical phenotype, the number of studies specifically addressing CKD was insufficient to allow a meaningful subgroup analysis. This reduces the ability to generalize our findings across distinct kidney disease settings and underscores the need for further research focused on soluble Fas in chronic kidney populations.

Recent clinical studies compellingly demonstrate that patients with kidney dysfunction exhibit significantly elevated levels of sFas and creatinine. Therefore, our findings reveal that sFas is a uremic retention solute in patients with kidney diseases. Notably, our research indicates that serum levels of sFas are directly correlated with those of the inflammatory cytokine IL-6. Elevated serum sFas levels have been consistently identified in patients with CKD and cardiovascular conditions^13^. Inflammation has an important role in this process^15,17,18,19,24^. While sFas is classified as a uremic retention solute, further preclinical and clinical studies are needed to establish its significance as a uremic toxin^1^. Alternatively, sFas has the potential to be a powerful prognostic marker for the progression of kidney dysfunction, particularly when tailored to the patient’s unique phenotype^23,25,26,27^.

In addition to investigating sFas as a potential uremic retention solute, this study also included traditional inflammatory markers—CRP and IL-6—to better characterize the interaction between inflammation and apoptosis in kidney dysfunction. While CRP and IL-6 are established indicators of systemic inflammation, sFas reflects apoptosis-related and uremia-specific mechanisms^28,29^. The combined analysis allowed a more integrated view of renal injury, indicating that sFas parallels the inflammatory response and mirrors the impaired clearance typical of uremic states^30,31^. Altogether, these findings highlight sFas as a promising biomarker and support the development of novel diagnostic and therapeutic tools applicable to clinical practice, with the potential to enhance early detection and targeted management of kidney disease^32,33^.

## Conclusion

This meta-analysis demonstrates that serum sFas levels are significantly higher in patients with kidney dysfunction. This association remained consistent despite moderate heterogeneity, and no relevant publication bias was detected. The combined analysis with CRP and IL-6 provided a broader understanding of the interplay between inflammation and apoptosis in renal injury. Elevated CRP and IL-6 levels correlated with increased sFas, reinforcing its link to systemic inflammation and impaired clearance in uremic states. Taken together, the results emphasize the potential of sFas as a relevant biomarker in kidney dysfunction and underline its contribution to the creation of innovative diagnostic and therapeutic approaches for clinical application, which should be further confirmed through large-scale studies.

## Data Availability

The data supporting the findings of this study will be publicly available at the time of publication in an online repository named Figshare, accessible via the following link: https://figshare.com/s/998bd555849273a1eb63.

## Supplementary Material

The following online material is available for this article:

Annex – Database Search Strategy with Field Tags.

Figure S1 – Funnel plot assessing publication bias in the included studies.

Table S1 – Summary of characteristics of studies included in the article.
