# Supplementary material for: Exploring the retention of soluble Fas protein in kidney dysfunction and its link to inflammation: a systematic review and meta-analysis
Source: J Bras Nefrol. 2026 Mar 9;48(2):e20250146. doi: 10.1590/2175-8239-JBN-2025-0146en (PMC12991439; doi:10.1590/2175-8239-JBN-2025-0146en)
Supplement: Supplementary file 1 [file 2175-8239-jbn-48-2-e20250146-suppl3.pdf]

Braz. J. Nephrol.
https://doi.org/10.1590/2175-8239-JBN-2025-0146en

Supplementary Material to "Exploring the retention of soluble Fas protein in kidney dysfunction and its link to inflammation: a systematic review and meta-analysis"

Table S1—Summary of characteristics of studies included in the article.

	PARTICIPANTS (N) AND CHARACTERISTICS	MEAN SFAS LEVELS (PG/ML) BETWEEN GROUPS		MEAN SERUM CREATININE LEVELS (MG/DL) BETWEEN GROUPS		MEAN IL-6 LEVELS (PG/ML) BETWEEN GROUPS		MEAN C-REACTIVE PROTEIN LEVELS (MG/DL) BETWEEN GROUPS		CLASSIFICATION OF THE RISK OF BIAS
		KIDNEY DYSFUNCTION	NO KIDNEY DYSFUNCTION	KIDNEY DYSFUNCTION	NO KIDNEY DYSFUNCTION	KIDNEY DYSFUNCTION	NO KIDNEY DYSFUNCTION	KIDNEY DYSFUNCTION	NO KIDNEY DYSFUNCTION	
**COHORT STUDIES**										
**GÓES et al. (2013)**	**n** = 72 (AKI group, n = 53; Non-AKI group, n = 19; Healthy controls, n = 18)<br>Percentage of males: AKI group, 64%; Non-AKI group, 63%; Healthy controls, 67%<br>Mean Age (years): AKI group, 62; Non-AKI group, 72; Healthy controls, 44	3885 ± 1878	Non-AKI: 1877 ± 1211<br>Controls: 1050 ± 295	2.90 ± 1.61	1.06 ± 0.46 / 0.99 ± 0.17	556 ± 587	57.1 ± 70.1 / 5.00 ± 6.81	-	-	Good quality
**GÓES et al. (2010)**	**n** = 110 (chronic kidney disease [CKD], n = 52; peritoneal dialysis [PD], n = 29; hemodialysis [HD], n = 29; control group, n = 29)<br>Percentage of males: CKD, 33%; PD, 12%; HD, 19%; Controls, 17%<br>Mean age (years): CKD, 57; PD, 54; HD, 47; Controls, 50	CKD: 3,121 ± 1,200<br>PD: 4,302 ± 1,225<br>HD: 4,608 ± 804	1,455 ± 844	-	-	7.36 ± 6.54* / 8.33 ± 7.07* / 7.06 ± 10.4	4.03 ± 5.63	0.90 ± 2.16 / 1.50 ± 2.54 / 1.48 ± 2.50	0.51 ± 0.64	Good quality
**PERIANAYAGAM et al. (2000)**	17 patients with chronic kidney disease (CKD); 11 patients with end-stage renal disease (ESRD); and 15 controls<br>Mean age (years): CKD, 58; ESRD, 53	CKD: 17,711 ± 1,177 pg/mL; HD: 23,505 ± 880 pg/mL; and PD (peritoneal dialysis): 29,011 ± 1,568 pg/mL	9,561 ± 503 pg/mL	CKD = 3.1 ± 0.6 mg/dL	Controls: 1.0 ± 0.1mg/dL;	-	-	-	-	Good quality

	PARTICIPANTS (N) AND CHARACTERISTICS	MEAN SFAS LEVELS (PG/ML) BETWEEN GROUPS		MEAN SERUM CREATININE LEVELS (MG/DL) BETWEEN GROUPS		MEAN IL-6 LEVELS (PG/ML) BETWEEN GROUPS		MEAN C-REACTIVE PROTEIN LEVELS (MG/DL) BETWEEN GROUPS		CLASSIFICATION OF THE RISK OF BIAS
		KIDNEY DYSFUNCTION	NO KIDNEY DYSFUNCTION	KIDNEY DYSFUNCTION	NO KIDNEY DYSFUNCTION	KIDNEY DYSFUNCTION	NO KIDNEY DYSFUNCTION	KIDNEY DYSFUNCTION	NO KIDNEY DYSFUNCTION	
**KORKES et al. (2013)**	**n** = 89 (AKI, 30; non-AKI, 13; ESRD on HD, 25; controls, 21)	AKI: 4,709 ± 2,562<br>ESRD: 4,806 ± 674	Non-AKI: 1,923 ± 1,207<br>Controls: 1,147± 369	AKI: 1.97 ± 0.84<br>ESRD: 8.57 ± 2.29	Non-AKI: 1.16 ± 0.63<br>Controls: 0.96 ± 0.16	AKI: 521 ± 583<br>ESRD: 6.61 ± 10.9	Non-AKI: 65.3 ± 69.9<br>Controls: 440 ± 6.38	-	-	Good quality
**SANO et al. (1998)**	**n** = 32 (Minimal change disease [MC], 4; Membranous nephropathy [MN], 3; Mesangial proliferative glomerulonephritis [mesPGN], 6; Membranoproliferative glomerulonephritis [MPGN], 1<br>Percentage of males: 43.75%<br>Mean age (years):<br>MC: 38 ± 16<br>MN: 49 ± 13<br>mesPGN: 42 ± 14<br>MPGN: 55 ± 16	mesPGN: 3,400 ± 900<br>MPGN: 3,900 ± 1,500<br>Mean: 3,650 ± 1,200	MC: 2,300 ± 600<br>MN: 2,200 ± 700<br>Mean: 2,250 ± 650	mesPGN: 1.2 ± 0.3<br>MPGN: 1.2 ± 0.2<br>Mean: 1.2 ± 0.25	MC: 0.8 ± 0.3<br>MN: 0.7 ± 0.1<br>Mean: 0.75 ± 0.2	mesPGN: 5,200 ± 6,100<br>MPGN: 7,400 ± 7,300* (*p < 0,05 vs. controls)	MC: 1,600 ± 2,900<br>MN: 2,300 ± 3,200	mesPGN: 0.19 ± 0.20<br>MPGN: 0.14 ± 0.13	MC: 0.06 ± 0.03<br>MN: 0.06 ± 0.05	Good quality
**SHOU et al. (1999)**	**n** = 58 (48 patients with IgA nephropathy; 10 patients with non-IgA PGN)<br>No additional demographic information.	Advanced-stage IgA nephropathy: 2.620 ± 180<br>Advanced-stage non-IgA PGN: 2.260 ± 120	Mild-stage IgA nephropathy: 1.790 ± 190<br>Mild-stage non-IgA PGN: 500 ± 0	Advanced-stage IgA nephropathy: 0.97 ± 0.07 mg/dL<br>Advanced-stage non-IgA PGN: 0.61 ± 0.07 mg/dL	Mild-stage IgA nephropathy: 0.77 ± 0.04 mg/dL<br>Mild-stage non-IgA PGN: 0.72 ± 0.06 mg/dL	-	-	-	-	Good quality
**ADLY et al. (2016)**	**n** = 35 (23 patients had Sickle cell disease [SCD]; 12 β-sickle cell thalassemia)<br>Percentage of males: 65.7%<br>Mean age (years): 8.4 ± 3.69 in SCD patients; 9.1 ± 3.2 in controls	1,900 (1,300–2,600)	1,400 (1,000–1,450)	-	-	-	-	-	-	Good quality
**BHATRAJU et al. (2017)**	**n** = 1,241 (Diabetes mellitus [DM], 28%; Chronic kidney disease [CKD], 9%; Sepsis, 58%; Septic shock, 20%)<br>Percentage of males: 65%<br>Mean age (years): 54 ±16	AKI Resolving: 11,586 (8,095–15,700)<br>AKI Nonresolving: 12,879 (8,938–17,682)	8,810 (6,880–11,926)	AKI Resolving: 2.0 ± 1.8<br>AKI Nonresolving: 2.2 ± 2.4	0.8 ± 0.4	AKI Resolving: 137 (59–351)<br>AKI Nonresolving: 147 (58–375)	75 (31–178)	-	-	Good quality
**DALBONI et al. (2003)**	**n** = 66 (Chronic kidney disease [CKD], 27; Hemodialysis, 14; Continuous peritoneal dialysis, 11; and Controls, 14)<br>Percentage of males: 51%<br>Mean age (years): 53.7	CKD: 1,696 ± 112<br>Hemodialysis: 1,756 ± 55<br>Continuous Peritoneal Dialysis: 1,599 ± 285	1,122 ± 262	CKD: 3.4 ± 2.7<br>Hemodialysis: 11.6 ± 3.3<br>Continuous Peritoneal Dialysis: 11 ± 3.4	-	-	-	CKD: 12 ± 23<br>Hemodialysis: 17 ± 48<br>Continuous Peritoneal Dialysis: < 0.8	< 0.8	Good quality

	PARTICIPANTS (N) AND CHARACTERISTICS	MEAN SFAS LEVELS (PG/ML) BETWEEN GROUPS		MEAN SERUM CREATININE LEVELS (MG/DL) BETWEEN GROUPS		MEAN IL-6 LEVELS (PG/ML) BETWEEN GROUPS		MEAN C-REACTIVE PROTEIN LEVELS (MG/DL) BETWEEN GROUPS		CLASSIFICATION OF THE RISK OF BIAS
		KIDNEY DYSFUNCTION	NO KIDNEY DYSFUNCTION	KIDNEY DYSFUNCTION	NO KIDNEY DYSFUNCTION	KIDNEY DYSFUNCTION	NO KIDNEY DYSFUNCTION	KIDNEY DYSFUNCTION	NO KIDNEY DYSFUNCTION	
**STÉPHAN et al. (2003)**	**n** = 107 (End stage renal disease secondary to chronic glomerulopathies, 26%; Diabetes Mellitus, 20%; and Hypertension, 12%; Nephropathies, Polycystic kidney disease, 16%; and Others, 26%) Percentage of males: 56.1% Mean age (years): 70	2,800 ± 940	-	-	-	-	-	11	-	Good quality
**NONOMURA et al. (2000)**	**n** = 47 (31 patients with Renal cell carcinoma; 8 patients with Benign prostatic hyperplasia; 4 Normal volunteers; 4 Female patients with stress incontinence; and 16 Controls)	3,640 ± 880	2,440 ± 670	-	-	-	-	-	-	Good quality
**EL-ADROUDY et al. (2000)**	**n** = 90 (30 Hemodialysis; 30 Chronic kidney disease [CKD]; 30 Controls) Percentage of males: 53.3% Mean age (years): 47	CKD: 23,100 ± 3,900 Hemodialysis: 12,500 ± 1,200	5,600 ± 1,300	CKD: 9.3 ± 1.41 Hemodialysis: 3.6 ± 1.23	0.7 ± 0.08	-	-	CKD: 6.9 ± 4.1 Hhemodialysis: 4.6 ± 2.7	1.1 ± 0.6	Good quality
**ZWIECH et al. (2013)**	84 patients with Primary glomerulonephritis Percentage of males: 53.6% Mean age (years): 41.44	12,100 ± 11,300	3,100 ± 1,700 pg/ml	-	-	-	-	-	-	Good quality
**CROSS-SECTIONAL STUDIES**										

	PARTICIPANTS (N) AND CHARACTERISTICS	MEAN SFAS LEVELS (PG/ML) BETWEEN GROUPS		MEAN SERUM CREATININE LEVELS (MG/DL) BETWEEN GROUPS		MEAN IL-6 LEVELS (PG/ML) BETWEEN GROUPS		MEAN C-REACTIVE PROTEIN LEVELS (MG/DL) BETWEEN GROUPS		CLASSIFICATION OF THE RISK OF BIAS
		**KIDNEY DYSFUNCTION**	**NO KIDNEY DYSFUNCTION**	**KIDNEY DYSFUNCTION**	**NO KIDNEY DYSFUNCTION**	**KIDNEY DYSFUNCTION**	**NO KIDNEY DYSFUNCTION**	**KIDNEY DYSFUNCTION**	**NO KIDNEY DYSFUNCTION**	
**MASRI et al. (2000)**	72 patients with Uremic syndrome and 11 Control patients<br>Percentage of males: 51.4%<br>Mean age (years): 3.57	Median: 5.7 (3.4-13.7) U/mL<br>Non-verotoxin hemorrhagic colitis: 6.4 (0-16.4) U/mL<br>Verotoxin hemorrhagic colitis: 10.3 (3.5-19.4) U/mL HUS	Median: 5.2 U/mL	-	-	-	-	-	-	Low risk
**DALBONI et al. (2008)**	25 patients with Terminal kidney disease; 27 patients with Chronic kidney disease; and 14 Controls<br>Percentage of males: 55.4%<br>Mean age (years):<br>60 in CDK patients and 61 in Controls	2,781 ± 1,214 pg/mL	2,196 ± 773 pg/mL	-	-	-	-	-	-	Low risk
**DOUNOUSI (2012)**	152 patients with Chronic kidney disease<br>Percentage of males: 55%<br>Mean age (years): 62	10,900 (± 275)	-	-	-	2.9 pg/mL	-	2.0 (mg/mL)	-	Low risk
**MORILLAS et al. (2012)**	159 Hypertensive patients<br>Percentage of males: 67.3%<br>Mean age (years): 56 ± 13	116.5 [84.4–137.4] pg/mL	105.3 [78.1–153.1] pg/mL	85 ± 18.7 mL/min	94.8 ± 18 mL/min	2.06 [1.6–2.91] pg/mL	1.6 [1.6–1.72] pg/mL	0.25 [0.12–0.59] mg/L	0.22 [0.1–0.39] mg/L	Low risk
**SATO et al. (2000)**	Chronic glomerulonephritis (CGN) group: 25 patients<br>Chronic renal failure (CRF) group: 40 patients<br>Hemodialysis (HD) group: 14 patients<br>Control group: 22 participants<br>Mean age (years):<br>CGN: 50 ± 14<br>CRF: 48 ± 21<br>HD: 64 ± 17<br>Controls: 47 ± 16	CGN (mild kidney dysfunction): 1.75 ± 0.94 ng/mL<br>CRF (chronic renal failure): 3.37 ± 1.29 ng/mL<br>HD (hemodialysis): 4.03 ± 0.65 ng/mL	Controls: 1.27 ± 0.46 ng/mL	CGN: < 2.0 mg/dL (by definition)<br><br>CRF: > 2.0 mg/dL	-	-	-	-		Low risk

	PARTICIPANTS (N) AND CHARACTERISTICS	MEAN SFAS LEVELS (PG/ML) BETWEEN GROUPS		MEAN SERUM CREATININE LEVELS (MG/DL) BETWEEN GROUPS		MEAN IL-6 LEVELS (PG/ML) BETWEEN GROUPS		MEAN C-REACTIVE PROTEIN LEVELS (MG/DL) BETWEEN GROUPS		CLASSIFICATION OF THE RISK OF BIAS
		KIDNEY DYSFUNCTION	NO KIDNEY DYSFUNCTION	KIDNEY DYSFUNCTION	NO KIDNEY DYSFUNCTION	KIDNEY DYSFUNCTION	NO KIDNEY DYSFUNCTION	KIDNEY DYSFUNCTION	NO KIDNEY DYSFUNCTION	
**TOMIYAMA et al. (2006)**	96 patients with Chronic kidney disease (CKD), pre-dialysis<br><br>Percentage of males: 67%<br><br>Mean age (years): Median 55 (range 20–69)	Kidney dysfunction (CACS > 0): 16.48 (2.12–43.01) mg/mL<br><br>Severe dysfunction (CACS > 400): 21.13 (4.17–43.01) mg/mL	No kidney dysfunction (CACS = 0): 12.74 (3.17–36.21) mg/mL	2.1 mg/dL (range: 0.9–5.6).	-	-	-	Kidney dysfunction (CACS > 0): 4.1 mg/L (range: 0.5–47.0).	No kidney dysfunction (CACS = 0): 3.9 mg/L (range: 0.1–22.6).	Low risk
**BABA et al. (2004)**	168 patients with Type II diabetes<br><br>Percentage of males: 56%<br><br>Mean age (years): 65	Stages 3B and 4 of diabetes, considering creatinine levels: 5,150 pg/mL	Stages 1, 2, and 3A of diabetes, considering creatinine levels: 2,700 pg/mL	Stages 3B and 4 of diabetes: 2.5 mg/dL	Stages 1, 2, and 3A of diabetes: 0.8 md/dL	-	-	Stages 3B and 4 of diabetes: 0.3 mg/dL	Stages 1, 2, and 3A of diabetes: 0.67 mg/dL	Low risk
**NIEWCZAS et al. (2008)**	Type 1 diabetes and normoalbuminuria patients (n = 363); microalbuminuria patients (n = 304)<br><br>Percentage of males: Normoalbuminuria, 40% Microalbuminuria, 61%<br><br>Mean age (years): Normoalbuminuria, 38.5 Microalbuminuria, 40.5	Normoalbuminuria: 4,500 pg/mL	Microalbuminuria: 4,950 pg/mL	-	-	-	-	0.115 mg/dL	0.15 mg/dL	Low risk

	PARTICIPANTS (N) AND CHARACTERISTICS	MEAN SFAS LEVELS (PG/ML) BETWEEN GROUPS		MEAN SERUM CREATININE LEVELS (MG/DL) BETWEEN GROUPS		MEAN IL-6 LEVELS (PG/ML) BETWEEN GROUPS		MEAN C-REACTIVE PROTEIN LEVELS (MG/DL) BETWEEN GROUPS		CLASSIFICATION OF THE RISK OF BIAS
		KIDNEY DYSFUNCTION	NO KIDNEY DYSFUNCTION	KIDNEY DYSFUNCTION	NO KIDNEY DYSFUNCTION	KIDNEY DYSFUNCTION	NO KIDNEY DYSFUNCTION	KIDNEY DYSFUNCTION	NO KIDNEY DYSFUNCTION	
**AMMIRATI et al. (2006)**	**n** = 49 (Hypertension, 20%; Diabetes, 20%; Chronic glomerulonephritis, 16%; Tubulointerstitial disease, 8%; Unknown, 36%) Percentage of males: 45% Mean age (years): Median 52 (range 20–70)	(score >10): 1,414 (842–2,997)	(score <10): 1,262 (519–2,631)	-	-	-	-	6.8 (0.3–67.5)	-	Low risk
