# Supplementary material for: Exploring the retention of soluble Fas protein in kidney dysfunction and its link to inflammation: a systematic review and meta-analysis
Source: J Bras Nefrol. 2026 Mar 9;48(2):e20250146. doi: 10.1590/2175-8239-JBN-2025-0146en (PMC12991439; doi:10.1590/2175-8239-JBN-2025-0146en)
Supplement: Supplementary file 2 [file 2175-8239-jbn-48-2-e20250146-suppl1.pdf]

Braz. J. Nephrol.
https://doi.org/10.1590/2175-8239-JBN-2025-0146en

Supplementary Material to "Exploring the retention of soluble Fas protein in kidney dysfunction and its link to inflammation: a systematic review and meta-analysis"

Annex - Database Search Strategy with Field Tags.

Database	Search Strategy
**MEDLINE**	#1 "sFas" OR "Soluble Fas" [MeSH] OR "Acute Kidney Injury" OR "Acute Kidney Disease" OR "Chronic Kidney Disease" OR "End Stage Kidney Disease" OR "Kidney" [MeSH] OR_"Outcomes" [MeSH]<br>(Fas Receptor / OR sFas.mp. OR CD95.mp. OR TNFRSF6.mp. OR (soluble.mp. AND (Fas.mp. OR CD95.mp.))) AND (Acute Kidney Injury / OR Renal Insufficiency, Chronic / OR Kidney Failure, Chronic / OR Kidney Diseases / OR AKI.mp. OR CKD.mp. OR ESRD.mp.) AND (Treatment Outcome / OR Prognosis / OR outcome*.mp. OR mortality.mp.)
**PubMed**	(Fas Receptor [MeSH] OR sFas [tiab] OR CD95 [tiab] OR TNFRSF6 [tiab] OR (soluble [tiab] AND (Fas [tiab] OR CD95 [tiab]))) AND (Acute Kidney Injury [MeSH] OR Renal Insufficiency, Chronic [MeSH] OR Kidney Failure, Chronic [MeSH] OR Kidney Diseases [MeSH] OR AKI [tiab] OR CKD [tiab] OR ESRD [tiab]) AND (Treatment Outcome [MeSH] OR Prognosis [MeSH] OR outcome*[tiab] OR mortality [tiab])
**SciELO**	("sFas" OR "soluble Fas" OR "CD95" OR "TNFRSF6") AND ("acute kidney injury" OR "chronic kidney disease" OR "end-stage renal disease" OR "kidney disease" OR AKI OR CKD OR ESRD) AND (outcome* OR prognosis OR mortality)

Notes - Synonyms were combined with OR and core concepts with AND; searches used MeSH terms, mp (Ovid MEDLINE multipurpose), and tiab (PubMed title/abstract).
