# Supplementary material for: Exploring the retention of soluble Fas protein in kidney dysfunction and its link to inflammation: a systematic review and meta-analysis
Source: J Bras Nefrol. 2026 Mar 9;48(2):e20250146. doi: 10.1590/2175-8239-JBN-2025-0146en (PMC12991439; doi:10.1590/2175-8239-JBN-2025-0146en)
Supplement: Supplementary file 4 [file 2175-8239-jbn-48-2-e20250146-suppl6.pdf]

Braz. J. Nephrol.
https://doi.org/10.1590/2175-8239-JBN-2025-0146pt

Material Suplementar para "Explorando a retenção da proteína Fas solúvel na disfunção renal e sua ligação com a inflamação: uma revisão sistemática e meta-análise"

Tabela S1—Resumo das características dos estudos incluídos na revisão.

	PARTICIPANTES (N) E CARACTERÍSTICAS	MÉDIA DOS NÍVEIS DE SFAS (PG/ML) ENTRE GRUPOS		MÉDA DO NÍVEL DE CREATININA SÉRICA (MG/DL) ENTRE GRUPOS		MÉDIA DO NÍVEL DE IL-6 (PG/ML) ENTRE GRUPOS		MÉDIA DE PROTEÍNA C-REATIVA (MG/DL) ENTRE GRUPOS		CLASSIFICAÇÃO DO RISCO DE VIÉS
		COM DISFUNÇÃO RENAL	SEM DISFUNÇÃO RENAL	COM DISFUNÇÃO RENAL	SEM DISFUNÇÃO RENAL	COM DISFUNÇÃO RENAL	SEM DISFUNÇÃO RENAL	COM DISFUNÇÃO RENAL	SEM DISFUNÇÃO RENAL	
**COHORT STUDIES**										
**GÓES et al. (2013)**	53 pacientes com injúria renal aguda (IRA); 19 sem IRA; e 18 controles<br>Porcentagem de homens: grupo IRA, 64%; grupo não-IRA, 63%; grupo controle, 67%<br>Idade média (anos): grupo IRA, 62; grupo não-IRA, 72; grupo controle, 44	3885 ± 1878	Grupo não-IRA: 1877 ± 1211<br>Grupo controle: 1050 ± 295	2,90 ± 1,61	Grupo não-IRA: 1,06 ± 0,46<br>Grupo controle: 0,99 ± 0,17	556 ± 587	Grupo não-IRA: 57,1 ± 70,1<br>Grupo controle: 5,00 ± 6,81	-	-	Boa qualidade
**GÓES et al. (2010)**	52 pacientes com doença renal crônica (DRC); 29 em diálise peritoneal (DP); 29 em hemodiálise (HD); 29 controles<br>Porcentagem de homens: DRC, 33%; DP, 12%; HD, 19%; Controles, 17%;<br>Idade média (anos): DRC, 57; DP, 54; HD, 47; Controles, 50	DRC: 3121 ± 1200<br>DP: 4302 ± 1225<br>HD: 4608 ± 804	1455 ± 844	-	-	7,36 ± 6,54* / 8,33 ± 7,07* / 7,06 ± 10,4	4,03 ± 5,63	0,90 ± 2,16 / 1,50 ± 2,54 / 1,48 ± 2,50	0,51 ± 0,64	Boa qualidade
**PERIANAYAGA M et al. (2000)**	17 pacientes com doença renal crônica (DRC); 11 pacientes com doença renal terminal; e 15 controles<br>Idade média (anos): DRC, 58; doença renal terminal, 53; controles, 44	DRC: 17711 ± 1177 pg/mL<br>HD: 23505 ± 880 pg/mL<br>Diálise peritoneal: 29011 ± 1568 pg/mL	9561 ± 503 pg/mL	DRC: 3,1 ± 0,6 mg/dL; doença renal terminal: NA	Controles: 1,0 ± 0,1mg/dL	-	-	-	-	Boa qualidade

	PARTICIPANTES (N) E CARACTERÍSTICAS	MÉDIA DOS NÍVEIS DE SFAS (PG/ML) ENTRE GRUPOS		MÉDA DO NÍVEL DE CREATININA SÉRICA (MG/DL) ENTRE GRUPOS		MÉDIA DO NÍVEL DE IL-6 (PG/ML) ENTRE GRUPOS		MÉDIA DE PROTEÍNA C-REATIVA (MG/DL) ENTRE GRUPOS		CLASSIFICAÇÃO DO RISCO DE VIÉS
		COM DISFUNÇÃO RENAL	SEM DISFUNÇÃO RENAL	COM DISFUNÇÃO RENAL	SEM DISFUNÇÃO RENAL	COM DISFUNÇÃO RENAL	SEM DISFUNÇÃO RENAL	COM DISFUNÇÃO RENAL	SEM DISFUNÇÃO RENAL	
**KORKES et al. (2013)**	89 pacientes (injúria real aguda, 30; não-IRA, 13; doença terminal renal com HD, 25; controles, 21)	IRA: 4709 ± 2562<br>Doença renal terminal: 4806 ± 674	não-IRA: 1923 ± 1207<br>Controles: 1147 ± 369	IRA: 1,97 ± 0,84<br>Doença renal terminal: 8,57 ± 2,29	não-IRA: 1,16 ± 0,63<br>Controles: 0,96 ± 0,16	IRA: 521 ± 583<br>Doença renal terminal: 6,61 ± 10,9	não-IRA: 65,3 ± 69,9<br>controles: 440 ± 6,38	-	-	Boa qualidade
**SANO et al. (1998)**	32 pacientes (Doença de Lesões Mínimas [DLM], 4; Nefropatia Membranosa [NM], 3; Glomerulonefrite Mesangial Proliferativa [GNPMe], 6; Glomerulonefrite Membranoproliferativa [GNMP], 1)<br>Porcentagem de homens: 54%<br>Idade média (anos):<br>DLM: 38 ± 16<br>NM: 49 ± 13<br>GNPMe: 42 ± 14<br>MPGN: 55 ± 16	GNPMe: 3400 ± 900<br>GNMP: 3900 ± 1500<br>Média: 3650 ± 1200	DLM: 2300 ± 600<br>NM: 2200 ± 700<br>Média: 2250 ± 650	GNPMe: 1,2 ± 0,3<br>GNMP: 1,2 ± 0,2<br>Média: 1,2 ± 0,25	DLM: 0,8 ± 0,3<br>NM: 0,7 ± 0,1<br>Média: 0,75 ± 0,2	GNPMe: 5200 ± 6100<br>GNMP: 7400 ± 7300* (*p < 0,05 vs. controles)	DLM: 1600 ± 2900<br>NM: 2300 ± 3200	GNPMe: 0,19 ± 0,20<br>GNMP: 0,14 ± 0,13	DLM: 0,06 ± 0,03<br>NM: 0,06 ± 0,05	Boa qualidade
**SHOU et al. (1999)**	58 pacientes (Nefropatia por IGA, 48; Glomerulonefrite proliferativa não-IgA, 10).<br>Sem outras informações demográficas.	Nefropatia por IgA avançada: 2,620 ± 180<br>Glomerulonefrite proliferativa não-IgA avançada: 2,260 ± 120	Nefropatia por IgA leve: 1,790 ± 190<br>Glomerulonefrite proliferativa não-IgA leve: 500 ± 0	Nefropatia por IgA avançada: 0,97 ± 0,07 mg/dL<br>Glomerulonefrite proliferativa não-IgA avançada: 0,61 ± 0,07 mg/dL	Nefropatia por IgA leve: 0,77 ± 0,04 mg/dL<br>Glomerulonefrite proliferativa não-IgA leve: 0,72 ± 0,06 mg/dL	-	-	-	-	Boa qualidade
**ADLY et al. (2016)**	35 pacientes (Anemia falciforme, 23; β-talassemia, 12)<br>Porcentagem de homens: 65,7%<br>Idade média (anos): 8,4 ± 3,69 entre pacientes com anemia falciforme; 9,1 ± 3,2 entre controles	1900 (1300–2600)	1400 (1000–1450)	-	-	-	-	-	-	Boa qualidade
**BHATRAJU et al. (2017)**	1241 pacientes com injúria renal aguda (IRA)<br>Porcentagem de homens: 65%<br>Idade média (anos): 54 ±16	IRA resolvendo: 11586 (8095–15700)<br>IRA não resolvendo: 12879 (8938–17682)	8810 (6880–11926)	IRA resolvendo: 2,0 ± 1,8<br>IRA não resolvendo: 2,2 ± 2,4	0,8 ± 0,4	IRA resolvendo: 137 (59–351)<br>IRA não resolvendo: 147 (58–375)	75 (31–178)	-	-	Boa qualidade

	PARTICIPANTES (N) E CARACTERÍSTICAS	MÉDIA DOS NÍVEIS DE SFAS (PG/ML) ENTRE GRUPOS		MÉDA DO NÍVEL DE CREATININA SÉRICA (MG/DL) ENTRE GRUPOS		MÉDIA DO NÍVEL DE IL-6 (PG/ML) ENTRE GRUPOS		MÉDIA DE PROTEÍNA C-REATIVA (MG/DL) ENTRE GRUPOS		CLASSIFICAÇÃO DO RISCO DE VIÉS
		**COM DISFUNÇÃO RENAL**	**SEM DISFUNÇÃO RENAL**	**COM DISFUNÇÃO RENAL**	**SEM DISFUNÇÃO RENAL**	**COM DISFUNÇÃO RENAL**	**SEM DISFUNÇÃO RENAL**	**COM DISFUNÇÃO RENAL**	**SEM DISFUNÇÃO RENAL**	
**DALBONI et al. (2003)**	27 pacientes com doença renal crônica (DRC), 14 em hemodiálise, 11 em diálise peritoneal contínua e 14 controles<br>Porcentagem de homens: 51%<br>Idade média (anos): 53,7	DRC: 1696 ± 112<br>Hemodiálise: 1756 ± 55<br>Diálise peritoneal contínua: 1599 ± 285	1122 ± 262	DRC: 3,4 ± 2,7<br>Hemodiálise: 11,6 ± 3,3<br>Diálise peritoneal contínua: 11 ± 3,4	-	-	-	DRC: 12 ± 23<br>Hemodiálise: 17 ± 48<br>Diálise peritoneal contínua: < 0,8	< 0,8	Boa qualidade
**STÉPHAN et al. (2003)**	107 pacientes com doença renal terminal (secundária a glomerulopatias crônicas, 26%; diabetes, 20%; hipertensão, 12%; doença policística renal, 16%; e outros, 26%)<br>Porcentagem de homens: 56,1%<br>Idade média (anos): 70	2800 ± 940	-	-	-	-	-	11	-	Boa qualidade
**NONOMURA et al. (2000)**	47 pacientes com doença renal;<br>31 pacientes com carcinoma de células renais;<br>9 homens com hiperplasia prostática benigna;<br>4 mulheres com incontinência por estresse;<br>4 pacientes saudáveis<br>Sem mais informações demográficas	3640 ± 880	2440 ± 670	-	-	-	-	-	-	Boa qualidade
**EL-ADROUDY et al. (2000)**	30 pacientes em hemodiálise; 30 pacientes com doença renal crônica (DRC); 30 controles<br>Porcentagem de homens: 53,3%<br>Idade média (anos): 47	DRC: 23100 ± 3900<br>Hemodiálise: 12500 ± 1200	5600 ± 1300	DRC: 9,3 ± 1,41<br>Hemodiálise: 3,6 ± 1,23	0,7 ± 0,08	-	-	DRC: 6,9 ± 4,1<br>Hemodiálise: 4,6 ± 2,7	1,1 ± 0,6	Boa qualidade

	PARTICIPANTES (N) E CARACTERÍSTICAS	MÉDIA DOS NÍVEIS DE SFAS (PG/ML) ENTRE GRUPOS		MÉDA DO NÍVEL DE CREATININA SÉRICA (MG/DL) ENTRE GRUPOS		MÉDIA DO NÍVEL DE IL-6 (PG/ML) ENTRE GRUPOS		MÉDIA DE PROTEÍNA C-REATIVA (MG/DL) ENTRE GRUPOS		CLASSIFICAÇÃO DO RISCO DE VIÉS
		**COM DISFUNÇÃO RENAL**	**SEM DISFUNÇÃO RENAL**	**COM DISFUNÇÃO RENAL**	**SEM DISFUNÇÃO RENAL**	**COM DISFUNÇÃO RENAL**	**SEM DISFUNÇÃO RENAL**	**COM DISFUNÇÃO RENAL**	**SEM DISFUNÇÃO RENAL**	
**ZWIECH et al. (2013)**	84 pacientes com glomerulonefrite primária<br>Porcentagem de homens: 53,6%<br>Idade média (anos): 41,44	12100 ± 11300	3100 ± 1700 pg/mL	-	-	-	-	-	-	Boa qualidade
**CROSS-SECTIONAL STUDIES**										
**MASRI et al. (2000)**	72 pacientes com síndrome urêmica e 11 controles<br>Porcentagem de homens: 51,4%.<br>Idade média (anos): 3,57	mediana: 5,7 (3,4-13,7) U/mL<br>Colite hemorrágica não verotóxica: 6,4 (0-16,4) U/mL<br>Colite hemorrágica verotóxica: 10,3 (3,5-19,4) U/mL SHU	mediana: 5,2 U/mL	-	-	-	-	-	-	Baixo risco
**DALBONI et al. (2008)**	25 pacientes com doença renal terminal; 27 pacientes com doença renal crônica; e 14 controles<br>Porcentagem de homens: 55,4%<br>Idade média (anos): 60	2781 ± 1214 pg/mL	2196 ± 773 pg/mL	-	-	-	-	-	-	Baixo risco
**DOUNOUSI (2012)**	152 pacientes com doença renal crônica<br>Porcentagem de homens: 55%<br>Idade média (anos): 62	10900 (± 275)	-	-	-	2,9 pg/mL	-	2,0 (mg/mL)	-	Baixo risco
**MORILLAS et al. (2012)**	159 pacientes hipertensos<br>Porcentagem de homens: 67,3%<br>Idade média (anos): 56 ± 13	116,5 [84,4–137,4] pg/mL	105,3 [78,1–153,1] pg/mL	85 ± 18,7 mL/min	94,8 ± 18 mL/min	2,06 [1,6–2,91] pg/mL	1,6 [1,6–1,72] pg/mL	0,25 [0,12–0,59] mg/L	0,22 [0,1–0,39] mg/L	Baixo risco

	PARTICIPANTES (N) E CARACTERÍSTICAS	MÉDIA DOS NÍVEIS DE SFAS (PG/ML) ENTRE GRUPOS		MÉDA DO NÍVEL DE CREATININA SÉRICA (MG/DL) ENTRE GRUPOS		MÉDIA DO NÍVEL DE IL-6 (PG/ML) ENTRE GRUPOS		MÉDIA DE PROTEÍNA C-REATIVA (MG/DL) ENTRE GRUPOS		CLASSIFICAÇÃO DO RISCO DE VIÉS
		COM DISFUNÇÃO RENAL	SEM DISFUNÇÃO RENAL	COM DISFUNÇÃO RENAL	SEM DISFUNÇÃO RENAL	COM DISFUNÇÃO RENAL	SEM DISFUNÇÃO RENAL	COM DISFUNÇÃO RENAL	SEM DISFUNÇÃO RENAL	
**SATO et al. (2000)**	Glomerulonefrite crônica: 25 pacientes<br>Insuficiência renal crônica: 40 pacientes<br>Hemodiálise: 14 pacientes<br>Grupo controle: 22 pacientes<br><br>Idade média (anos):<br>Glomerulonefrite crônica, 50 ± 14;<br>Insuficiência renal crônica, 48 ± 21;<br>Hemodiálise, 64 ± 17;<br>Controles, 47 ± 16	Glomerulonefrite crônica: 1,75 ± 0,94 ng/mL<br><br>Insuficiência renal crônica: 3,37 ± 1,29 ng/mL<br><br>Hemodiálise: 4,03 ± 0,65 ng/mL	Controles: 1,27 ± 0,46 ng/mL	Glomerulonefrite crônica: < 2,0 mg/dL (por definição)<br><br>Insuficiência renal crônica: > 2,0 mg/dL	-	-	-	-		Baixo risco
**TOMIYAMA et al. (2006)**	96 pacientes com doença renal crônica pré-diálise<br><br>Porcentagem de homens: 67%<br><br>Idade média (anos): 55 (variação 20–69)	Disfunção renal (Escore de Cálcio Coronário > 0): 16,48 (2,12–43,01) mg/mL<br><br>Disfunção severa (Escore de Cálcio Coronário > 400): 21,13 (4,17–43,01) mg/mL	Sem disfunção renal (Escore de Cálcio Coronário = 0): 12,74 (3,17–36,21) mg/mL	2,1 mg/dL (variação: 0,9–5,6).	-	-	-	Disfunção renal (Escore de Cálcio Coronário > 0): 4,1 mg/L (variação: 0,5–47,0).	Sem disfunção renal (Escore de Cálcio Coronário = 0): 3,9 mg/L (variação: 0,1–22,6)	Baixo risco
**BABA et al. (2004)**	Paciente com diabetes tipo 2 (n = 168)<br><br>Porcentagem de homens: 56%<br><br>Idade média (anos): 65	Diabetes estágios 3B e 4, considerando níveis de creatinina: 5150 pg/mL	Diabetes estágios 1, 2 e 3A, considerando níveis de creatinina: 2700 pg/mL	Diabetes estágios 3B e 4: 2,5 mg/dL	Diabetes estágios 1, 2 e 3A: 0,8 mg/dL	-	-	Diabetes estágios 3B e 4: 0,3 mg/dL	Diabetes estágios 1, 2 e 3A: 0,67 mg/dL	Baixo risco

	PARTICIPANTES (N) E CARACTERÍSTICAS	MÉDIA DOS NÍVEIS DE SFAS (PG/ML) ENTRE GRUPOS		MÉDA DO NÍVEL DE CREATININA SÉRICA (MG/DL) ENTRE GRUPOS		MÉDIA DO NÍVEL DE IL-6 (PG/ML) ENTRE GRUPOS		MÉDIA DE PROTEÍNA C-REATIVA (MG/DL) ENTRE GRUPOS		CLASSIFICAÇÃO DO RISCO DE VIÉS
		COM DISFUNÇÃO RENAL	SEM DISFUNÇÃO RENAL	COM DISFUNÇÃO RENAL	SEM DISFUNÇÃO RENAL	COM DISFUNÇÃO RENAL	SEM DISFUNÇÃO RENAL	COM DISFUNÇÃO RENAL	SEM DISFUNÇÃO RENAL	
**NIEWCZAS et al. (2008)**	Pacientes com diabetes tipo 1 e normoalbuminúria (n = 363) ou microalbuminúria (n = 304)<br>Porcentagem de homens: Normoalbuminúria, 40%;<br>Microalbuminúria, 61%<br>Idade média (anos): Normoalbuminúria, 38,5;<br>Microalbuminúria: 40,5	Normoalbuminúria: 4500 pg/mL	Microalbuminúria: 4950 pg/mL	-	-	-	-	0,115 mg/dL	0,15 mg/dL	Baixo risco
**AMMIRATI et al. (2006)**	49 pacientes<br>Porcentagem de homens: 45%<br>Idade média (anos): 52 (variação: 20–70)<br>Diagnóstico–Causas de doença renal crônica:<br>Hipertensão, 20%; Diabetes, 20%; Glomerulonefrite crônica, 16%; Doença tubulointersticial, 8%; Desconhecido, 36%	(pontuação > 10): 1.414 (842–2.997)	(pontuação < 10): 1.262 (519–2.631)	-	-	-	-	6,8 (0,3–67,5)	-	Baixo risco
