# Supplementary material for: Exploring the retention of soluble Fas protein in kidney dysfunction and its link to inflammation: a systematic review and meta-analysis
Source: J Bras Nefrol. 2026 Mar 9;48(2):e20250146. doi: 10.1590/2175-8239-JBN-2025-0146en (PMC12991439; doi:10.1590/2175-8239-JBN-2025-0146en)
Supplement: Supplementary file 5 [file 2175-8239-jbn-48-2-e20250146-suppl4.pdf]

Braz. J. Nephrol.
https://doi.org/10.1590/2175-8239-JBN-2025-0146pt

Material Suplementar para “Explorando a retenção da proteína Fas solúvel na disfunção renal e sua ligação com a inflamação: uma revisão sistemática e meta-análise”

Anexo—Estratégia de busca em bases de dados com etiquetas de campo.

Base de dados	Estratégia de Busca
**MEDLINE**	#1 “sFas” OU “Fas Solúvel” [MeSH] OU “Lesão Renal Aguda” OU “Doença Renal Aguda” OU “Doença Renal Crônica” OU “Doença Renal Terminal” OU “Rim” [MeSH]_OU “Desfechos” [MeSH].<br><br>(Receptor Fas / OU sFas.mp. OU CD95.mp. OU TNFRSF6.mp. OU (soluble.mp. AND (Fas.mp. OU CD95.mp.))) E (Lesão Renal Aguda / OU Insuficiência Renal, Crônica / OU Falência Renal, Crônica / OU Doenças Renais / OU LRA.mp. OU DRC.mp. OU DRT.mp.) E (Desfechos de Tratamento / OU Prognóstico / OU resultado*.mp. OU mortalidade.mp.).
**PubMed**	(Receptor Fas [MeSH] OU sFas [tiab] OU CD95 [tiab] OU TNFRSF6 [tiab] OU (solúvel [tiab] E (Fas [tiab] OU CD95 [tiab]))) E (Lesão Renal Aguda [MeSH] OU Insuficiência Renal, Crônica [MeSH] OU Falência Renal, Crônica [MeSH] OU Doenças Renais [MeSH] OU LRA [tiab] OU DRC [tiab] OU DRT [tiab]) E (Desfechos de Tratamento [MeSH] OU Prognóstico [MeSH] OU resultado*[tiab] OU mortalidade[tiab]).
**SciELO**	(“sFas” OU “Fas solúvel” OU “CD95” OU “TNFRSF6”) E (“lesão renal aguda” OU “doença renal crônica” OU “doença renal terminal” OU “doença renal” OU LRA OU DRC OU DRT) E (resultado* OU prognóstico OU mortalidade).

Notas - Sinônimos foram combinados com OU e os conceitos principais com E; as buscas usaram termos MeSH, mp (campo multipropósito do MEDLINE) e tiab (título/resumo do PubMed).
