# Supplementary material for: Exploring the retention of soluble Fas protein in kidney dysfunction and its link to inflammation: a systematic review and meta-analysis
Source: J Bras Nefrol. 2026 Mar 9;48(2):e20250146. doi: 10.1590/2175-8239-JBN-2025-0146en (PMC12991439; doi:10.1590/2175-8239-JBN-2025-0146en)
Supplement: Supplementary file 6 [file 2175-8239-jbn-48-2-e20250146-suppl5.pdf]

Braz. J. Nephrol.
https://doi.org/10.1590/2175-8239-JBN-2025-0146pt

Material Suplementar para “Explorando a retenção da proteína Fas solúvel na disfunção renal e sua ligação com a inflamação: uma revisão sistemática e meta-análise”

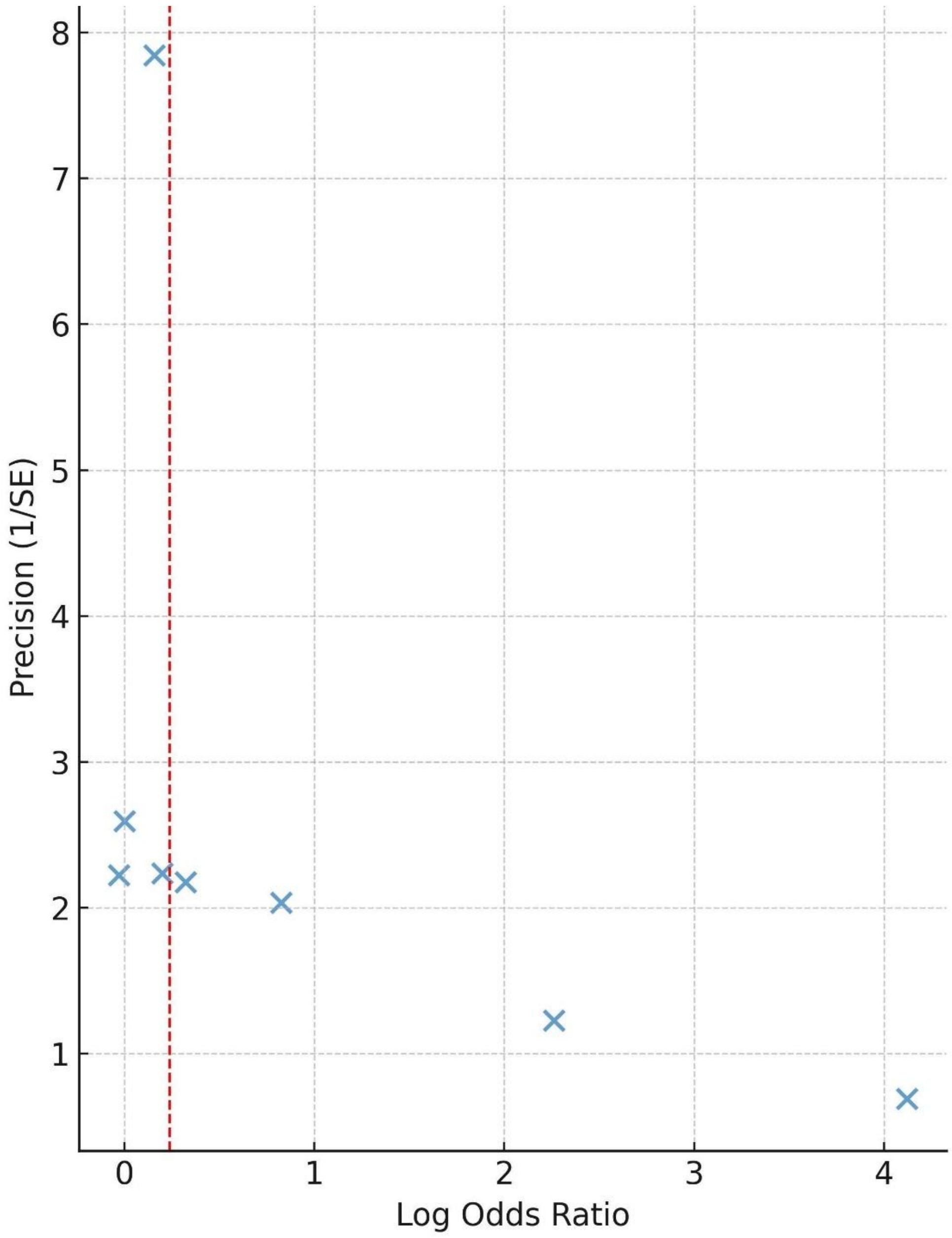

Abreviaturas - *SE, *standard error* / erro padrão.

Figura S1 - Gráfico de funil avaliando o viés de publicação nos estudos incluídos.
