# Supplementary material for: Exploring the retention of soluble Fas protein in kidney dysfunction and its link to inflammation: a systematic review and meta-analysis
Source: J Bras Nefrol. 2026 Mar 9;48(2):e20250146. doi: 10.1590/2175-8239-JBN-2025-0146en (PMC12991439; doi:10.1590/2175-8239-JBN-2025-0146en)
Supplement: Translation

# Translation: Explorando a retenção da proteína Fas solúvel na disfunção renal e sua ligação com a inflamação: uma revisão sistemática e meta-análise

**DOI:** 10.1590/2175-8239-JBN-2025-0146pt

**Authors:** Beatriz Moreira Silva, Giovana Irikura Cardoso, Priscila Giusti Lázaro, Bárbara Formaggio Domingues, Isabele Pardo, Carolina Sponton, Miguel Angelo Goes

**Affiliations:** 1 Escola Paulista de Medicina, Universidade Federal de São Paulo, São Paulo, SP, Brasil.; 2 Faculdade Israelita de Ciências da Saúde Albert Einstein, São Paulo, SP, Brasil.

**Keywords:** Doença Renal Crônica, Lesão Renal Aguda, Fas Solúvel, Inflamação, Soluto Urêmico

## Abstract

**Introdução:**

De acordo com estudos recentes, os níveis séricos de Fas solúvel (sFas) estão relacionados a marcadores inflamatórios, doenças cardiovasculares, anemia e disfunção renal. O presente estudo analisa a associação entre o sFas, marcadores inflamatórios e disfunção renal.

**Métodos:**

Esta meta-análise foi realizada utilizando o programa R, juntamente com uma revisão sistemática que empregou a escala Newcastle-Ottawa e as orientações do *Joanna Briggs Institute* para avaliar o risco de viés em estudos observacionais. Foram utilizadas as bases de dados eletrônicas PubMed, MEDLINE e SciELO. Filtros de pesquisa foram utilizados para incluir todos os artigos em inglês. A heterogeneidade foi avaliada utilizando o teste Q de Cochran, τ^2^ (tau-quadrado) e a estatística I^2^, e um modelo de efeitos aleatórios (método *DerSimonian-Laird*) foi aplicado devido à presença de heterogeneidade moderada entre os estudos de coorte incluídos.

**Resultados:**

A revisão sistemática e a meta-análise compreenderam 24 artigos, que apresentaram um risco significativo de viés. Analisando os estudos de coorte selecionados, 1.449 pacientes apresentavam disfunção renal. Destes, 512 (57%) eram homens. Os valores médios de creatinina sérica, IL-6, proteína C reativa e sFas (8,635 pg/mL, 3,206 pg/mL) foram mais elevados em pacientes com disfunção renal. Também houve correlações positivas entre os níveis séricos de sFas, IL-6 e creatinina. Isso sugere um papel potencial do sFas na inflamação e na progressão das doenças renais.

**Conclusão:**

Nosso estudo confirmou e demonstrou que o sFas é um soluto de retenção urêmica associado à inflamação. São necessárias mais pesquisas para confirmar o sFas como biomarcador ou seu papel como toxina urêmica.

## Introdução

A disfunção renal, tanto na lesão renal aguda (LRA) quanto na doença renal crônica (DRC), é caracterizada pela diminuição da função renal e pelo aumento dos níveis de solutos de retenção urêmica^1,2,3,4^.

O Fas solúvel (sFas) é uma proteína derivada do receptor CD95+ (mFas)^5^. Ao contrário do receptor CD95+, que está ancorado na membrana celular, o sFas não possui um domínio transmembranar devido à clivagem da metaloproteinase do mFas ou ao *splicing* alternativo do pré-mRNA CD95+. Enquanto o mFas funciona como uma glicoproteína transmembranar e ativa a apoptose e outras vias, através do ligante Fas (FasL), o sFas pode interferir na sinalização do mFas^5,6,7,8,9,10,11^.

Níveis elevados de sFas sérico estão associados a doenças cardiovasculares, anemia e inflamação na DRC^12,13,14,15,16,17,18^. Os níveis séricos de sFas também se correlacionam significativamente com anemia e inflamação na LRA^19,20^. A relação crítica entre o sFas e a inflamação na disfunção renal pode auxiliar os profissionais de saúde e pesquisadores a compreender e desenvolver estratégias de tratamento eficazes para essas condições graves.

Reconhecendo que a inflamação influencia as doenças cardiovasculares e a anemia na DRC e na LRA, realizamos este estudo para compreender melhor a relação potencial entre o sFas, os marcadores da função renal e a inflamação na disfunção renal, tais como a proteína C reativa (PCR) e a interleucina-6 (IL-6).

## Métodos

Este estudo é uma revisão sistemática e meta-análise. Realizamos uma pesquisa exaustiva nas plataformas PubMed, MEDLINE e SciELO utilizando termos MeSH (Material Suplementar). Os autores selecionaram cuidadosamente os artigos a serem incluídos no estudo, seguindo critérios rigorosos. Artigos experimentais, meta-análises e artigos de revisão foram excluídos. Em contrapartida, foram incluídos artigos publicados em inglês que utilizavam termos MeSH e filtros de pesquisa para estudos observacionais (Anexo Suplementar). A última pesquisa foi realizada em 20 de janeiro de 2024.

Utilizamos a *Newcastle-Ottawa Scale* (NOS) para avaliar e filtrar o risco de viés nos estudos de coorte incluídos nesta revisão sistemática. A NOS avalia a qualidade de estudos não randomizados com base na seleção, comparabilidade e análise de resultados. De um total de 21 artigos de coorte, apenas 14 foram selecionados para a análise e, entre eles, um artigo foi classificado como de baixa qualidade e removido do estudo, resultando em 13 artigos de coorte selecionados para a revisão sistemática. Para estudos transversais (n = 13), utilizamos a lista de verificação do *Joanna Briggs Institute* (JBI), uma ferramenta validada para estudos observacionais. Dois estudos foram excluídos devido ao alto risco de viés, restando 11 estudos transversais para análise. Após triagem e avaliação de elegibilidade, 24 estudos atenderam aos critérios de inclusão e foram selecionados para análise (Figura 1).

**Figura 1:** Resumo da pesquisa e seleção de evidências.

O risco de viés e a qualidade das evidências foram avaliados independentemente por dois revisores. Quaisquer divergências foram resolvidas por consenso com um terceiro revisor. As avaliações da qualidade e do risco de viés orientaram diretamente a inclusão e a interpretação dos estudos: apenas estudos de coorte com pontuações NOS moderadas a altas e estudos transversais classificados como de baixo risco de viés pelo JBI foram mantidos para síntese. Essas avaliações não foram aplicadas apenas como ferramentas de triagem, mas também utilizadas para contextualizar a força das evidências nas seções Resultados e Discussão.

Um total de 13 estudos foi incluído na revisão sistemática. No entanto, a síntese quantitativa foi restrita a 8 estudos de coorte, uma vez que eles forneceram dados estatisticamente comparáveis sobre a associação entre sFas, creatinina sérica e marcadores de inflamação, propondo relações entre essas variáveis. Os demais estudos transversais foram revisados descritivamente e excluídos da análise combinada para minimizar inconsistências metodológicas.

Todas as análises estatísticas foram realizadas no software R (versão 4.1.2) usando os pacotes “meta” e “metafor”. Um modelo de efeitos aleatórios (REM) foi empregado para explicar a variabilidade entre as investigações, garantindo que os pesos dos estudos fossem calculados de acordo com a variância, em vez de distribuídos igualmente. Os tamanhos dos efeitos foram expressos como *odds ratios* (OR) ou diferenças médias padronizadas (DMP), ambos com intervalos de confiança (ICs) de 95%.

Além disso, a heterogeneidade foi avaliada utilizando o teste Q de Cochran (χ^2^, com p < 0,10 indicando heterogeneidade), o índice de inconsistência (I^2^, expresso como a porcentagem da variabilidade total não atribuível ao acaso) e τ^2^ (estimativa da variância entre estudos). Essas medidas, juntamente com os tamanhos dos efeitos combinados e os pesos dos estudos, são explicitamente apresentadas nos gráficos de floresta. Ainda, para explorar o potencial viés de publicação, geramos um gráfico em funil (Figura Suplementar 1). Na síntese quantitativa principal dos oito estudos de coorte, foi aplicado o teste de Egger; no entanto, os resultados foram interpretados com cautela, dado o número limitado de artigos. Análises de subgrupos por fenótipo clínico (DRC vs. LRA) foram consideradas, mas não realizadas, pois o pequeno número de revisões em cada categoria não permitiria comparações robustas.

Cada estudo contribuiu com um peso estatístico distinto para a estimativa combinada, de acordo com seu tamanho amostral e variância, conforme exibido no gráfico de floresta. Esse gráfico também apresenta as estimativas individuais das investigações com ICs de 95%, juntamente com os pesos atribuídos sob o modelo de efeitos aleatórios.

De acordo com a Resolução CNS 510/2016 (Seção IV), pesquisas realizadas exclusivamente com textos científicos para uma revisão da literatura científica não são registradas nem avaliadas pelo sistema de comitês de ética (CEP/CONEP). Assim, o presente estudo pode ser realizado sem aprovação e processamento locais. Mesmo assim, indexamos nosso projeto no PROSPERO, um respeitado banco de dados internacional para protocolos de revisão sistemática, reforçando a transparência metodológica.

## Resultados

Realizamos a seleção dos artigos de forma ética e sistemática (Figura 1). Inicialmente, havia 8.517 pesquisas. Após uma triagem rigorosa, remoção de duplicatas e revisão por pares, selecionamos 965 artigos. Para refinar ainda mais essa seleção, excluímos os estudos que não foram publicados em inglês e aqueles que não atendiam aos nossos critérios de inclusão predefinidos. Embora tenhamos eliminado pesquisas que não atendiam aos requisitos de desenho de estudo, ensaios clínicos randomizados ou subanálises apropriadas, garantimos que apenas pesquisas relevantes e de alta qualidade fossem incluídas.

Isso resultou na exclusão de 941 artigos. No entanto, 24 foram considerados os mais relevantes para inclusão na revisão sistemática e meta-análise, compreendendo 13 estudos de coorte de boa qualidade e 11 estudos transversais com baixo risco de viés. A NOS e a orientação do JBI foram utilizadas não apenas para a avaliação da qualidade metodológica, mas também desempenharam um papel fundamental na orientação da interpretação dos resultados da revisão. Nosso estudo começou com uma análise detalhada do risco de viés em estudos de coorte (Tabela 1) e estudos transversais (Tabela 2). Era essencial manter a integridade do nosso processo de revisão. Os dados dos estudos de coorte nos permitiram correlacionar diversas variáveis com grupos de pacientes, tanto aqueles com disfunção renal quanto aqueles sem. Notavelmente, 64% dos indivíduos analisados nos estudos apresentavam algum grau de disfunção renal. Entre os indivíduos incluídos, 836 (57%) eram homens, e a idade média dos pacientes com disfunção renal — um fator crítico no seu tratamento — era de 46 anos.

**Tabela 1:** Risco de viés dos estudos de coorte

Autores	Seleção				Comparabilidade	Desfecho		
	Representatividade da coorte exposta	Seleção da coorte não exposta	Confirmação da exposição	Demonstração de que o desfecho de interesse não estava presente no início do estudo	Comparabilidade das coortes com base no desenho do estudo ou na análise controlada para fatores de confusão	Avaliação do desfecho	O acompanhamento foi longo o suficiente para que os desfechos ocorressem	Adequação do acompanhamento das coortes
GÓES et al. (2013)^1^	*	*	*	*	**	*	*	*
GÓES et al. (2010)^21^	*	*	*	*	*	*	*	*
PERIANAYAGAM et al. (2000)^2^	*	*	*	*	**	*	*	*
KORKES et al. (2013)^9^	*	*	*	*	*	*	*	*
SANO et al. (1998)^11^	*	*	*	*	**	*	*	*
SHOU et al. (1999)^10^	*	*	*	*	**	*	–	*
ADLY et al. (2016)^22^	*	*	*	*	**	*	–	*
BHATRAJU et al. (2017)^13^	*		*	*	**	*	–	*
DALBONI et al. (2003)^16^	*	*	*	*	**	*	*	*
TROYANOV et al. (2003)^23^	*		*	*	*	–	*	*
NONOMURA et al. (2000)^24^	*	*		*	*	*	*	*
EL-ADROUDY et al. (2000)^8^	*	*	*	*	**	*	*	–
ZWIECH et al. (2013)^25^		*	*	*	**	*	*	*

**Tabela 2:** Risco de viés dos estudos transversais

Autores	Os critérios de inclusão na amostra foram claramente definidos?	Os participantes do estudo e o contexto foram descritos em detalhe?	A exposição foi mensurada de maneira válida e confiável?	Foram utilizados critérios objetivos e padronizados para a mensuração da condição?	Os fatores de confusão foram identificados?	As estratégias para lidar com os fatores de confusão foram descritas?	Os desfechos foram mensurados de maneira válida e confiável?	Foi utilizada análise estatística apropriada?
MASRI et al. (2000)^15^	S	S	S	S	S	S	S	S
DALBONI et al. (2008)^5^	S	S	S	S	S	S	S	S
DOUNOUSI et al. (2012)^18^	S	S	S	S	S	S	S	S
MORILLAS et al. (2012)^26^	S	S	S	S	S	S	S	S
SATO et al. (2000)^27^	S	S	S	S	S	S	S	S
TOMIYAMA et al. (2006)^30^	S	S	S	S	S	S	S	S
BABA et al. (2004)^28^	S	S	S	S	N	S	S	S
D’ANDREA et al. (2020)^32^	S	S	S	S	S	N	S	S
RESCH et al. (2021)^33^	S	S	S	S	S	N	S	S
NIEWCZAS et al. (2008)^29^	S	S	S	S	S	S	S	S
AMMIRATI et al. (2006)^31^	S	S	S	S	S	S	S	S

Em relação aos dados séricos, os pacientes com disfunção renal apresentaram um nível médio de creatinina sérica de 2,8 mg/dL, enquanto aqueles sem disfunção renal apresentaram um nível médio de 0,7 mg/dL. Também obtivemos valores séricos de sFas, constatando que o valor sérico médio para pacientes com disfunção renal foi de 8,635 pg/mL, em comparação com 3,206 pg/mL no grupo sem disfunção renal. O valor médio de PCR sérico para o grupo com envolvimento renal foi de 0,035 mg/dL, o que não diferiu significativamente do valor de 0,034 mg/dL observado no grupo de participantes do estudo sem disfunção renal. Além disso, os níveis séricos médios de IL-6 estavam notavelmente elevados em pacientes com disfunção renal (Tabela 3).

**Tabela 3:** Informações gerais de todos os estudos de coorte

Informações gerais	Envolvimento renal	Envolvimento não-renal
**Pacientes**	1449	815
**Mulher (%)**	512 (43)	365 (44)
**Homem (%)**	836 (57)	459 (56)
**Idade média dos pacientes**	46	35
**Creatinina sérica média (mg/dL)**	2,8	0,7
**sFas sérica média (pg/mL)**	8635	3206
**PCR sérica média (mg/dL)**	0,0359	0,0345
**IL-6 sérica média (pg/mL)**	193,4	29,7

Abreviaturas – sFas: Fas Solúvel; PCR: Proteína C Reativa; IL-6: Interleucina-6.

Notas – Os valores foram calculados a partir de dados extraídos de estudos previamente publicados^1,2,8,9,10,11,13,16,21,22,23,24,25^.

Um total de 13 estudos de coorte foram incluídos na revisão sistemática. Destes, apenas oito foram elegíveis para a meta-análise, por relatarem dados comparáveis e estatisticamente significativos sobre a associação entre os níveis de sFas e a disfunção renal. Os onze estudos transversais foram sintetizados narrativamente devido à heterogeneidade metodológica. Na síntese quantitativa dos oito estudos de coorte, os pacientes com função renal comprometida apresentaram níveis circulantes significativamente mais elevados de sFas em comparação com os controles (OR combinado = 1,27; IC 95% = 1,02–1,58; p = 0,03). A heterogeneidade entre os estudos foi moderada (Q de Cochran = 14,2; p = 0,048; I^2^ = 56%; τ^2^ = 0,0017), conforme demonstrado no gráfico de floresta (Figura 2).

**Figura 2:** Gráfico de floresta da associação entre sFas e creatinina.

Abreviaturas – OR: razão de chances; IC 95%: intervalo de confiança de 95%; I^2^: índice de inconsistência (heterogeneidade entre estudos); τ^2^: variância entre estudos (tau-quadrado); p: valor de significância estatística.

Marcadores inflamatórios, particularmente a IL-6, foram relatados em seis estudos. Embora algumas análises individuais tenham sugerido uma correlação positiva entre IL-6 e sFas, apenas duas investigações forneceram estimativas quantitativas, que foram insuficientes para permitir uma análise conjunta.

O viés de publicação foi avaliado para os oito estudos de coorte. A inspeção visual do gráfico em funil (Figura Suplementar 1) revelou simetria aproximada, sem evidências significativas de efeitos de estudos pequenos. O teste de regressão de Egger (p = 0,12) não indicou viés de publicação significativo; no entanto, a interpretação deve ser cautelosa, dado o número limitado de revisões (<10 estudos).

Nenhuma análise adicional de sensibilidade ou subgrupo (por exemplo, DRC vs. LRA) foi realizada devido ao pequeno número de estudos disponíveis em cada categoria. Essa limitação é reconhecida, pois a combinação de dados de DRC e LRA pode reduzir a interpretabilidade específica do fenótipo.

Em nossa análise estatística, extraímos informações sobre a relação entre sFas e o marcador inflamatório PCR. A meta-análise demonstrou uma associação estatisticamente significativa entre os valores de sFas e PCR em pacientes com disfunção renal [OR = 0,72; IC 95% = 0,70–0,73; p = 0,001]. No entanto, apenas três estudos forneceram dados suficientes para este cálculo, limitando a robustez e a generalização deste resultado.

## Discussão

Nossa pesquisa estabelece uma ligação clara entre os níveis séricos de sFas e os níveis de creatinina. Como a creatinina sérica é um marcador vital para avaliar a função renal em pacientes com doenças renais, é evidente que o sFas funciona como um soluto de retenção urêmica. Além disso, encontramos uma correlação significativa entre o sFas e os níveis séricos de IL-6, uma citocina pró-inflamatória importante. Nossas descobertas facilitam a compreensão da disfunção renal e fornecem informações valiosas para melhorar os esforços de pesquisa e as estratégias de tratamento de pacientes com complicações relacionadas à disfunção renal.

Analisamos treze estudos de coorte que atendiam a critérios de seleção elevados, cada um consistindo em coortes bem definidas e abrangentes. Notavelmente, o grupo sem disfunção renal foi extraído da mesma coorte de pesquisa que aqueles com LRA ou DRC, aumentando a confiabilidade de nossos resultados e contribuindo significativamente para o campo.

Os critérios essenciais de avaliação foram claramente definidos para garantir a comparabilidade adequada dos dados, mantendo a consistência na forma como os dados foram apresentados nos artigos. Enquanto alguns estudos se concentraram exclusivamente em subgrupos específicos, outros não esclareceram se o grupo sem disfunção renal foi explicitamente concebido para esse estudo, ao contrário do grupo com disfunção renal. No entanto, essas inconsistências não afetaram significativamente a qualidade geral dos resultados. Onze estudos transversais foram incluídos de forma descritiva devido à heterogeneidade metodológica, enquanto a síntese quantitativa foi restrita aos oito estudos de coorte que relataram dados comparáveis e estatisticamente significativos.

Abordamos a heterogeneidade por meio de testes estatísticos padrão e aplicamos um modelo de efeitos aleatórios, o que permitiu estimativas combinadas mais robustas. Embora o teste de Egger tenha sido interpretado com cautela, dado o número limitado de investigações, o gráfico em funil sugeriu um baixo risco de viés de publicação. Cada estudo foi ponderado pelo método da variância inversa, garantindo que dados mais precisos contribuíssem proporcionalmente para o efeito geral.

Por outro lado, onze estudos transversais foram selecionados com base na abrangência e qualidade das informações relativas aos critérios de inclusão. O processo de seleção foi realizado de forma aleatória, considerando fatores como a relevância do tema, uma associação clara entre sFas e disfunção renal por meio de objetivos bem definidos, medição adequada da exposição, rigor das análises estatísticas e outras considerações metodológicas relevantes.

No presente trabalho, quando analisamos estudos de pacientes com disfunção renal — seja LRA ou DRC — observamos que os níveis séricos de sFas eram mais elevados em pacientes com disfunção renal em comparação com indivíduos sem disfunção renal. Nosso grupo havia relatado anteriormente que os níveis séricos de sFas se correlacionam negativamente com a depuração da creatinina em pacientes com DRC e são mais elevados em pacientes com DRC avançada que necessitam de diálise de manutenção^21^. Um estudo de Dalboni et al. descobriu que os níveis séricos de sFas eram significativamente mais elevados em indivíduos com disfunção renal do que naqueles sem disfunção renal. Pacientes com DRC e doença cardiovascular apresentaram níveis ainda mais elevados de sFas. Houve uma correlação inversa entre os níveis séricos de sFas, a depuração da creatinina e a dosagem de eritropoetina humana recombinante. Além disso, os níveis de sFas apresentaram uma relação positiva com os níveis de PCR. Esses achados destacam a potencial retenção de solutos de sFas na disfunção renal, sugerindo que o sFas pode ser um biomarcador para inflamação e condições relacionadas à doença^16^.

Nossa análise identificou uma associação estatisticamente significativa entre os níveis séricos de sFas e PCR em pacientes com disfunção renal. No entanto, esse resultado foi baseado em dados extraídos de apenas três estudos, o que limita a força e a generalização da descoberta^16,21,22^. Também encontramos diferenças significativas nas análises dos níveis de IL-6 nos artigos que evidenciaram que os níveis séricos de IL-6 eram mais elevados em pacientes com LRA e DRC^19,20,23^. Os níveis séricos de IL-6 mostraram uma tendência de aumento com a deterioração da função renal^24^. Isso também sugere que o sFas sérico poderia ser um marcador-chave de inflamação em pacientes urêmicos. Apesar de vários estudos terem abordado os marcadores inflamatórios, com especial atenção para a IL-6, a disponibilidade limitada de dados quantitativos impediu uma análise estatística combinada. Embora alguns resultados tenham sugerido uma possível associação positiva entre a IL-6 e o sFas, essa relação deve ser considerada preliminar e geradora de hipóteses.

Nossos estudos anteriores demonstraram que os níveis séricos de sFas são mais elevados em pacientes com DRC do que naqueles sem disfunção renal^15,17,18,19,20,21^. Essa tendência também é evidente em pacientes com LRA, que apresentam níveis séricos de sFas mais elevados do que aqueles sem disfunção renal^19,20^. Além disso, foi encontrada uma forte conexão entre os níveis de sFas, inflamação e anemia em doenças renais. Como os níveis séricos de sFas são mais elevados em pacientes com disfunção renal do que em indivíduos saudáveis, isso destaca a importância do sFas como um potencial soluto de retenção urêmica.

Este estudo apresenta diversas limitações. Em primeiro lugar, faltaram artigos que investigassem explicitamente a relação entre o sFas e a disfunção renal em estudos de coorte e transversais. Embora as pesquisas incluídas tenham fornecido dados valiosos para analisar os perfis clínicos de pacientes com doenças renais, o número limitado de revisões sobre esse tema dificulta nossa capacidade de estabelecer correlações robustas e significativas entre as variáveis. Em segundo lugar, a interpretação do teste de regressão de Egger foi limitada pelo pequeno número de estudos incluídos, uma vez que esse método requer uma amostra maior para detectar com confiabilidade o viés de publicação. A análise da IL-6 também foi limitada pelo pequeno número de estudos que relataram esse marcador, reduzindo a robustez dos resultados. Por fim, incluímos as populações com LRA e DRC em uma análise combinada. Apesar de termos tentado estratificar os resultados por fenótipo clínico, o número de investigações que abordavam especificamente a DRC foi insuficiente para permitir uma análise de subgrupos significativa. Isso reduz a capacidade de generalizar nossos resultados em diferentes contextos de doença renal e ressalta a necessidade de mais pesquisas focadas no Fas solúvel em populações com doença renal crônica.

Estudos clínicos recentes demonstram de forma convincente que pacientes com disfunção renal apresentam níveis significativamente elevados de sFas e creatinina. Portanto, nossos resultados revelam que o sFas é um soluto de retenção urêmica em pacientes com doenças renais. Notavelmente, nossa pesquisa indica que os níveis séricos de sFas estão diretamente correlacionados com os da citocina inflamatória IL-6. Níveis séricos elevados de sFas têm sido consistentemente identificados em pacientes com DRC e condições cardiovasculares^13^. A inflamação tem um papel importante nesse processo^15,17,18,19,24^. Embora o sFas seja classificado como um soluto de retenção urêmica, são necessários mais estudos pré-clínicos e clínicos para estabelecer sua importância como toxina urêmica^1^. Alternativamente, o sFas tem o potencial de ser um poderoso marcador prognóstico para a progressão da disfunção renal, particularmente quando adaptado ao fenótipo único do paciente^23,25,26,27^.

Além de investigar o sFas como um possível soluto urêmico de retenção, o presente trabalho também incluiu marcadores inflamatórios tradicionais — PCR e IL-6 — para caracterizar melhor a interação entre inflamação e apoptose na disfunção renal. Enquanto a PCR e a IL-6 são indicadores consagrados de inflamação sistêmica, o sFas reflete mecanismos relacionados à apoptose e específicos do estado urêmico^28,29^. A análise combinada permitiu uma visão mais integrada da lesão renal, indicando que o sFas não apenas acompanha a resposta inflamatória, mas também reflete a depuração prejudicada típica das condições urêmicas^30,31^. Em conjunto, esses achados destacam o sFas como um biomarcador promissor e sustentam o desenvolvimento de novas ferramentas diagnósticas e terapêuticas aplicáveis à prática clínica, com potencial para aprimorar a detecção precoce e o manejo direcionado das doenças renais^32,33^.

## Conclusão

Esta meta-análise demonstra que os níveis séricos de sFas estão significativamente aumentados em pacientes com disfunção renal. Essa associação permaneceu consistente mesmo com heterogeneidade moderada, sem viés de publicação relevante. A análise conjunta com PCR e IL-6 ampliou a compreensão da interação entre inflamação e apoptose na lesão renal. Níveis elevados desses marcadores correlacionaram-se ao aumento de sFas, reforçando seu vínculo com a inflamação sistêmica e a depuração comprometida em estados urêmicos. Por fim, os resultados reforçam o potencial do sFas como um biomarcador relevante na disfunção renal e evidenciam sua contribuição para a criação de abordagens diagnósticas e terapêuticas inovadoras com aplicação clínica, as quais devem ser confirmadas em estudos de maior escala.

## Disponibilidade de Dados

Os dados que sustentam os achados deste estudo estarão disponíveis publicamente no momento da publicação em um repositório online chamado *Figshare*, por meio do link https://figshare.com/s/998bd555849273a1eb63.

## Material Suplementar

O seguinte material online está disponível para o presente artigo:

Anexo – Estratégia de busca em bases de dados com etiquetas de campo.

Figura S1 – Gráfico de funil avaliando o viés de publicação nos estudos incluídos.

Tabela S1 – Resumo das características dos estudos incluídos na revisão.
